# An artificial selection procedure enriches for known and suspected chitin degraders from the prokaryotic rare biosphere of multiple marine biotopes

**DOI:** 10.1186/s12866-025-04218-7

**Published:** 2025-11-25

**Authors:** Laurence Meunier, Tina Keller-Costa, David Cannella, Jorge M.S. Gonçalves, Etienne Dechamps, Matilde Marques, Rodrigo Costa, Isabelle F. George

**Affiliations:** 1 Laboratory of Ecology of Aquatic Systems, Brussels Bioengineering School, Université Libre de Bruxelles (ULB), Brussels, Belgium; 2 Institute for Bioengineering and Biosciences (iBB) and Institute for Health and Bioeconomy (i4HB), Instituto Superior Técnico (IST), Universidade de Lisboa, Lisbon, Portugal; 3 Department of Bioengineering, Instituto Superior Técnico (IST), Universidade de Lisboa, Lisbon, Portugal; 4 PhotoBioCatalysis Unit, Crop Nutrition and Biostimulation Lab (CPBL) and Biomass Transformation Lab (BTL), Brussels Bioengineering School, Université Libre de Bruxelles, Brussels, Belgium; 5 Algarve Centre of Marine Sciences (CCMAR), Universidade Do Algarve (UALG), Faro, Portugal; 6 Department of Biology, Laboratory of Marine Biology, Université Libre de Bruxelles, Brussels, Belgium

**Keywords:** Prokaryotic communities, Enrichment cultures, Chitinase, Marine sponge, Octocoral, Size exclusion chromatography

## Abstract

**Background:**

Biological chitin degradation is a major process in the ocean, governed primarily by the action of microorganisms. It is known that the structure and taxonomic profile of chitin-degrading microbial communities change across marine biotopes, but efforts to isolate chitin degraders within these communities in the laboratory have seldom been attempted. We characterized the prokaryotic communities associated with the marine sponge *Sarcotragus spinosulus*, the octocoral *Eunicella labiata*, and their surrounding sediment and seawater and applied an artificial selection procedure to enrich bacterial consortia capable of degrading chitin from the abovementioned biotopes. Throughout the procedure, chitin degradation was monitored, and the taxonomic composition was studied along four successive enrichment cultures from each biotope.

**Results:**

The naturally occurring prokaryotic communities of the two host species (*Sarcotragus spinosulus* and *Eunicella labiata*) were distinct from each other and from those of seawater and sediments, even though they were co-inhabiting the same geographic area. We found that low-abundance bacteria from the rare biosphere were recruited in the enrichment cultures from all biotopes, while dominant bacterial symbionts likely to play a role in chitin degradation within marine sponges and octocorals remained “unculturable” under our experimental conditions. Well-known chitin degraders such as *Vibrio*, *Pseudoalteromonas* and *Aquimarina*, as well as other taxa not known or poorly known for their role(s) in chitin degradation such as *Aureivirga*, *Halodesulfovibrio*, *Motilimonas*, *Muricauda*, *Psychromonas*, *Poseidonibacter*, *Reichenbachiella*, and *Thalassotalea*, among others, were enriched using our artificial selection approach. Distinct chitin-degrading consortia were enriched from each marine biotope, highlighting the feasibility of this approach in fostering the discovery of novel microorganisms and enzymes involved in chitin degradation pathways of relevance in applied biotechnology.

**Conclusion:**

This study unveils distinct bacterial consortia possessing moderate to high efficiency at degrading chitin. They were composed of a mix of known chitin degraders, known chitin utilizers and many taxa poorly or not yet known for their role(s) in chitin degradation such as *Aureivirga*, *Psychromonas, Motilimonas, Reichenbachiella, or Halodesulfovibrio*. The latter taxa are potential key players in marine chitin degradation whose study could lead to the discovery of novel enzyme variants able to degrade chitin and its derivatives.

**Supplementary Information:**

The online version contains supplementary material available at 10.1186/s12866-025-04218-7.

## Introduction

Chitin is the most abundant biopolymer in the marine environment, and chitin degradation is a major process dictating the cycling of carbon and nitrogen in the ocean [1]. Importantly, chitin does not accumulate in the ocean [2] but undergoes a rapid turnover due to the activity of chitin-degrading microorganisms. Chitin degradation can be mediated by autochthonous marine microorganisms [1] attached to chitinous exoskeletons or particulate detritus in the seawater column [3] or living in symbiosis with macroeukaryotic hosts such as crustaceans [1, 4], marine sponges [5] and octocorals [5, 6]. Chitin degrading bacteria are also found in large numbers in marine sediment [7]. Microbial chitin degradation leads to the production of small organic molecules such as N-acetylglucosamine (GlcNAc), glucosamine (GlcN), acetate, glucose (Glc) and deacetylated forms of chitin such as chitosan [8, 9]. These chitin degradation products can be assimilated by bacteria for cell material synthesis or mineralized into CO_2_ and NH_4_^+^ [9]. Microorganisms that directly act on the chitin polymer through the action of endochitinases or deacetylases are usually referred to as “chitin degraders”, whereas microorganisms which consume chitin degradation products such as chitooligosaccharides (COSs) and chitosan, through the action of exo-chitinases and chitosanases are referred to as “chitin utilizers” or “consumers” [9]. Endo- and exochitinases degrade chitin through a chitinolytic pathway, meaning that they hydrolyze the glycosidic bonds between the GlcNAc units of the chitin polymers (endochitinase) or of the COSs (exochitinases) [9].

Currently, molecular-based evidence suggests that distinct marine biotopes host taxonomically divergent chitin-degrading communities [5, 10, 11]. This indicates that a multitude of chitin degrading enzymes and accessory proteins (e.g., endochitinases, exochitinases, deacetylases, Lytic Polysaccharides MonoOxygenases (LPMOs), chitin-binding modules) with distinct substrate affinities and physical–chemical optima, produced by diverse chitin-degrading bacteria, remain unexplored and unknown. Current knowledge of the chitin degradation potential within symbiotic communities of sessile marine invertebrates is growing, as recent hypotheses have been raised on the roles of canonical symbionts of octocorals (*Endozoicomonadaceae*) [6] and marine sponges (*Rhodothermales*) [12] in the cycling of chitin in benthic ecosystems. However, despite the chitin catalytic potential existing within marine microbial communities, studies designed to harness the metabolism of chitinolytic marine consortia in the laboratory have seldom been attempted [13, 14]. Artificial selection (i.e., the making of enrichment cultures from environmental samples) is a powerful technique that allows to select communities efficient at degrading complex compounds such as pollutants or natural polymers like cellulose or chitin [14–17]. It consists of utilizing a natural prokaryotic community as inoculum in a specific medium (e.g., a medium with chitin as the sole source of carbon and nitrogen). Then, successive transfers to fresh culture medium help to progressively select a microbial community specialized in a particular biochemical process or pathway (e.g., chitin degradation). We posit that artificial selection procedures may increase the discoverability of chitin-degrading enzymes and organisms, holding promise for diverse industrial sectors. For example, chitinolytic enzymes can be used to create natural fungicides and insecticides that undermine the cell wall of fungi and the exoskeleton of insects [18–20]. They can also be used to generate COSs and chitosan from chitinous waste in a more ecofriendly manner than the conventional industrial processes based on concentrated acids and strong alkali at high temperature [21–24]. Those COSs and chitosan products bear potential for applications in various fields such as agriculture, water treatment, medicine, textiles, and more [18, 19].

In this study, we describe the prokaryotic community structures of a model High Microbial Abundance sponge (*Sarcotragus spinosulus*) and of a model octocoral (*Eunicella labiata*) species co-inhabiting the same geographic location (Algarve coast, Portugal), and those of their surrounding seawater and sediments (hereafter denoted “biotopes”), and assess their suitability as sources of novel chitin-degrading and utilizing bacteria. To this end, we employed an artificial selection procedure to enrich chitin-degrading consortia from these four marine biotopes and examined whether contrasting biotopes lead to taxonomically distinct chitin-degrading communities in the laboratory. During the artificial selection process, chitin degradation was assessed by measuring the change in molecular weight of the chitin polymer by Size Exclusion Chromatography (SEC) instead of using commercial kits that measure a potential degradation activity on short oligomers. Finally, we examined whether chitin-degrading microorganisms recruited during the artificial selection procedure correspond to so-far unknown microbial lineages taking part in chitin/chitin-derivative degradation processes, and whether they represent dominant or otherwise low-abundant (“rare”) organisms in the original communities. This study highlights putative novel chitinolytic bacteria by artificial selection from multiple marine biotopes.

## Material and methods

### Sampling

*Sarcotragus spinosulus* (Schmitt, 1862; Porifera, Demospongiae, Keratosa, Irciniidae) (three biological replicates: SP1, SP2, SP3) and *Eunicella labiata* (Thomson, 1927; Cnidaria, Anthozoa, Octocorallia, Eunicellidae) (three biological replicates: OC1, OC2, OC3) specimens and their surrounding seawater and sediment (three biological replicates each: SW1, SW2 and SW3 for seawater and SD1, SD2, SD3 for sediment) were collected off the Algarve coast, southern Portugal (“Pedra da Greta”: Lat. 36° 58′47.2N, Long. 7° 59′ 20.8W) at a depth of 18–19 m (bottom water pH was 8.13, temperature 19 °C, and salinity 36.41 ppt) on the 29th of September 2020 by scuba diving. Pieces of marine sponge and branches of octocoral specimens (about 5 g each) were cut with a sterile scalpel and placed individually with surrounding seawater into Ziploc® bags. Surface sediment was sampled with a sterile spoon (*c.* 2 g/replicate) at *c.* 1 m distance to the animals and kept in sterile pots. Finally, seawater samples (*c.* 2 L/replicate) were collected *c.* 1 m above the animals and stored in sterile bottles. Samples were transported to the laboratory in a cooling box (c. 30 min transport time) and sample processing started immediately upon arrival in the laboratory.

### Sample processing

In a laminar hood, marine sponges were handled with sterilized tweezers to remove macroscopic epibionts and extracellular endobionts such as mussels, gastropods, worms, and algae. Afterwards, marine sponge specimens were washed with sterile Artificial Seawater (ASW) (ASW: 23.38 g L − 1 NaCl, 2.41 g L − 1 MgSO_4_ ∗ 7H2O, 1.90 gL − 1 MgCl_2_ ∗ 6H_2_O, 1.11 g L − 1 CaCl_2_ ∗ 2H_2_O, 0.75 g L − 1 KCl and 0.17 g L − 1 NaHCO_3_) and cut into small pieces with a sterile scalpel. The octocoral branches were also checked for epibionts (which, if present, were removed), washed with sterile ASW and the tissue was then scraped off the internal, scleroprotein (“gorgonin”)-based skeleton of the samples with the help of a sterile scalpel and cut into small pieces. Several replicates of 0.25 g of tissue of each marine sponge and octocoral specimen were stored in sterile 2.0 mL microcentrifuge tubes at −80 °C until DNA extraction. Seawater samples (c. 500 mL) were filtered through 0.22 μm pore-size nitrocellulose membranes (Millipore, MERCK) with the help of a vacuum pump. Seawater filters and sediment samples (0.25 g/replicate) were stored in sterile 2.0 mL microcentrifuge tubes at −80 °C until DNA extraction.

Microbial cell pellets were obtained from 1 g of marine sponge and octocoral tissue according to the method described by [25] with minor modifications, as well as from 1 L of seawater and 1 g of sediment. The protocols to obtain the pellets are detailed in Supplementary File S1. Each cell pellet (seawater, sediment, marine sponge, and octocoral) was resuspended in 830 µL of sterile ASW and transferred into sterile, 2 mL cryo-vials equipped with 150 µL of sterile 100% glycerol and 20 µL of pure, 100% DMSO. These glycerol stocks were stored at −80 °C until further use.

### Artificial selection procedure

Glycerol stocks of microbial cell suspensions from the four biotopes (marine sponge, octocoral, sediment and seawater; three biological replicates each) were used as the starting material for artificial selection of microbial communities through successive transfers of enrichment cultures in a chitin-containing culture medium (described below). In total, twelve artificial selection experiments were initiated in this study.

**Fig. 1:**
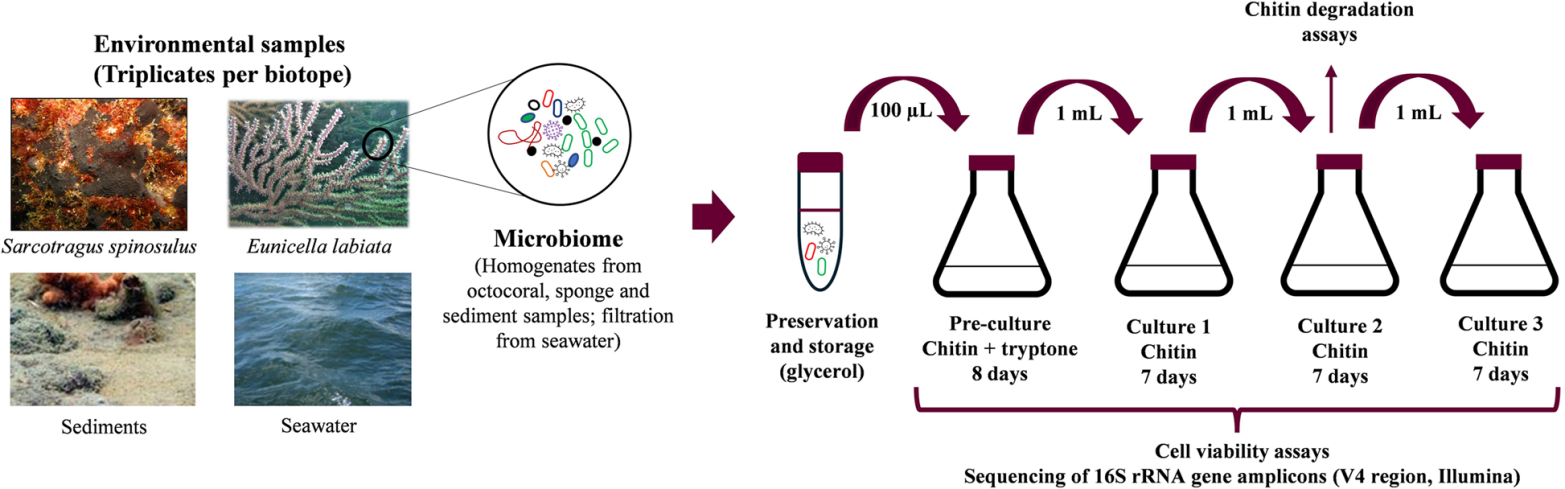
Experimental design of the artificial selection process. SEC, Size Exclusion Chromatography. Image created with BioRender.com

The experimental setup for artificially selecting microbial communities is depicted in Fig. 1. The artificial selection process consisted of one pre-culture (referred to as “enrichment culture PC”) and three successive cultures (so-called “enrichment cultures C1, C2 and C3”). Briefly, 100 μL of each glycerol stock was inoculated into 100 mL of pre-culture medium at 20 °C and 85 rpm. After 8 days of incubation, 1 mL of this PC was added to 100 mL of enrichment culture medium. After 7 days of incubation, 1 mL of enrichment culture C1 was transferred to 100 mL of the same fresh enrichment culture medium (C2). This step was repeated once more to generate enrichment culture C3. The culture medium for the preparation of the enrichment cultures (C1, C2 and C3) was composed of 100 mL of autoclaved ASW, 0.15 g of KH_2_PO_4_, 1 g of chitin powder extracted from shrimp shells (C7170 from Sigma-Aldrich/MERCK, Germany) and 160 µL of a solution of trace elements (for details, see Table S1). The same medium was amended with 0.01 g of tryptone in the preparation of the pre-culture medium (PC) to boost the growth of bacteria at the beginning of the process (Fig. 1).

After each incubation period, the viability of the enrichment cultures was directly assessed using the MTT cell viability assay (detailed in Supplementary File S1). Briefly, the MTT viability assay is a colorimetric method used to measure cell metabolic activity and indirectly assess cell viability. In addition, the following samples were collected: i) 5 mL for semi-quantitative assessment of chitin degradation (as described below; only for enrichment cultures C2) followed by qualitative characterization of the chitin degradation products by size exclusion chromatography (SEC; only for enrichment culture C2) after storage of the samples at −80 °C, and ii) 5 mL for DNA extraction after centrifugation (10,000 g for 10 min) and storage of the pellet at −20 °C.

### Chitin degradation assessments

Assessments of chitin degradation were performed on the enrichment cultures C2 of each artificial selection experiment (in triplicates). Indeed, chitin was usually observed to be more effectively degraded in C2 cultures, as indicated by preliminary measurements of the amount of decanted chitin left in culture flasks by the end of each enrichment culture C1, C2 and C3.

Two approaches were used to assess chitin degradation: the measurement of remaining chitin weight in the cultures and size exclusion chromatography (SEC). Those techniques are complementary since measuring chitin weight constitutes an easy albeit not very precise approach, being explored in a semi-quantitative fashion in this study, while SEC provides qualitative information on the average size of the chitin polymer in the enrichment cultures (a decrease in polymer size indicates chitin degradation) and the size variations within the cultures (an increase in size variation suggests that the chitin polymer has been degraded into a range of diverse sizes).

First, to record the weight of chitin remaining in the enrichment cultures at the end of the C2 incubation period (semi-quantitative assessment), the chitin powder in the liquid culture (5 mL) was first centrifuged at 2,000 g for 5 min and the pellet washed twice with MilliQ water. The chitin pellet was then dried at 70 °C in heating blocks (DRB200; Hach, USA) until reaching a constant weight and weighted. The chitin weight loss for each enrichment culture C2 was calculated by subtracting the remaining chitin weight from the initial chitin mass, dividing by the initial mass, and then multiplying by 100.

Second, for a detailed assessment of chitin degradation dynamics, SEC was used to determine the molecular mass parameters of the chitin polymers: (i) numbered average molecular weight (Mn), defined as total weight of polymer divided by the total number of molecules; (ii) weight average molecular weight (Mw), which depends on the number of molecules present and on the weight of each molecule; and (iii) polydispersity (PDI), defined as the ratio of the weight average molecular weight to the number average molecular weight, giving a measure of the distribution of the molecular weight within a sample [26], with three technical replicates conducted for each sample. The molecular mass parameters of the chitin polymers were also determined for the negative control (C-; in triplicate) and were calculated as detailed in Supplementary File S1. The SEC protocol was applied to dry chitin pellets obtained from 5 mL of the C2 enrichment cultures and is described in detail in Supplementary File S1 (Fig. S1).

Finally, estimates of chitin weight loss and Mn_1_ values (“numbered average molecular weight” given by SEC) calculated as described above were integrated to classify the chitin degradation efficiency of samples analyzed in this study as “high”, “good”, “moderate” and “low” (see Table S2 for details).

### Total community DNA extraction and 16S rRNA gene sequencing

DNA was directly extracted from 0.25 g of the inner marine sponge tissue, octocoral tissue, sediment and from membranes on which seawater had been filtered for the environmental (in situ) samples; and from the microbial cell pellets recovered from 5 mL of each enrichment culture (PC, C1, C2 and C3). DNA was extracted using the “DNeasy PowerSoil Pro kit” from Qiagen (ID: 47,014) according to the manufacturer’s protocol. The filters were cut into small pieces using sterile scissors prior to DNA extraction. The DNA quantity (ng/µL) and quality (A260/A280 and A260/A230) was estimated with a NanoDrop ND 2000 UV–VIS spectrophotometer (Thermo Fisher Scientific, Waltham, US) and DNA samples were kept at −20 °C until further analyses. 16S rRNA gene amplification and sequencing from DNA samples were carried out at StarSeq (Mainz, Germany) using Illumina MiSeq sequencing. The V4 region of the 16S rRNA gene was sequenced following a 2 × 300 paired-end approach using the primers 515 F (5’-GTG YCA GCM GCC GCG GTAA-3’) and 806 Rb (5’-GGA CTA CNV GGG TWT CTA AT-3’) [27, 28]. An average of 100,000 paired-end sequences was generated per sample. The library of reads was demultiplexed by StarSeq.

### Processing of the 16S rRNA gene sequencing data

All 16S rRNA gene fragment sequences were processed using standard procedures within DADA2 (Divisive Amplicon Denoising Algorithm) v.1.8 (in R; [29]). Briefly, all raw reads with Ns were removed, bacteriophage PhiX control reads [30] were filtered out, and primer sequences were removed by trimming at the 5’ -end of each read. Forward and reverse reads were truncated to 240 nt (forward) and 150 nt (reverse) and filtered using default parameters. Error rates were computed to identify unique sequences, forward and reverse reads were merged, and chimeras were filtered out of the final ASV table (see Supplementary File S1 for details). Taxonomy of each ASV was then assigned using the SILVA database version 138.1 [31, 32].

The ASVs versus samples table and taxonomy profile table were then imported into R as a phyloseq object using the phyloseq package (v1.38.0; [33]) to perform diversity and taxonomic composition analyses. The dataset was filtered using the *subset_taxa* function within phyloseq to eliminate mitochondria, chloroplast and eukaryote sequences. The final dataset consisted of 60 samples (all sample types included) thoroughly profiled via 16S rRNA gene sequencing. A total of 5,685,383 filtered reads were generated and 5,389 bacterial and 284 archaeal ASVs were found.

### Analysis of 16S rRNA gene fragments

Data wrangling and visualization were performed in R using *phyloseq* (v1.38.0; [33]), *dplyr* (v1.8.6; [34]), and *ggplot2* (v3.4.0; [35]). Briefly, stacked bar charts were used to illustrate taxonomic composition at the phylum, class, and ASV levels. Alpha diversity was evaluated using observed ASV richness and the Shannon–Wiener index. Statistical differences between groups were tested via ANOVA, repeated measure ANOVA, and post-hoc (pairwise) tests. Beta diversity analyses compared prokaryotic community structures among environmental samples (seawater, sediment, marine sponge, octocoral) and between enrichment cultures derived from each biotope and from each biotope replicate. ASV data were Hellinger-transformed, then Bray–Curtis similarity matrices were calculated and used for Principal Coordinates Analysis (PCoA). Significant differences in community structure of environmental samples and/or enrichment cultures were looked for using PERMANOVA or Welch MANOVA, depending on variance homogeneity. For a detailed description of the methodology employed in the analysis of 16S rRNA gene profiles, see Supplementary File S1.

### Genome-wide inspection of chitin degradation and utilization features among artificially enriched and poorly studied bacterial genera

We searched the NCBI Genome Database (https://www.ncbi.nlm.nih.gov/datasets/genome/) for representative genomes of bacterial genera that were dominant in the enrichment cultures but remain poorly characterized or unknown in the context of chitin metabolism. Those genera were: *Motilimonas, Pseudophaeobacter, Reichenbachiella, Halodesulfovibrio, Aureivirga, Epibacterium, Psychromonas, Muricauda, Poseidonibacter,* and *Thalassotalea*. We analyzed whether those genomes contained genes involved in chitin catabolism to hypothesize their potential roles in chitin degradation and utilization. Our approach consisted of thorough protein family (Pfam) annotation of all genomes available on DOE JGI’s Integrated Microbial Genomes & Microbiomes (IMG/M) data management system v.7 [36] for the bacterial taxa under inspection. Specifically, we scanned 102 genomes from ten artificially-selected genera for the presence of 15 Pfam categories representing protein domains involved in hydrolysis of the large chitin polymer (endo-chitinases of GH families 18 and 19—EC 3.2.1.14, chitin-binding proteins), hydrolysis of chitin non-reducing ends (exo-chitinases, EC 3.2.1.52), chitin deacetylation (polysaccharide deacetylases), and N-acetylglucosamine binding and utilization.

## Results

### Prokaryotic community structure in environmental samples

Observed ASV richness and Shannon diversity indices varied significantly across biotopes (*S. Spinosulus*, *E. labiata*, seawater, and sediment) (ANOVA, *p* < 0.05; File S1, Fig. S2). Host-associated prokaryotic communities were less rich and diverse than those in seawater and sediment (Tukey test, *p* < 0.05). While the marine sponge and octocoral samples had similar ASV counts (Tukey test, *p* > 0.05), the Shannon index of sponge communities was significantly higher than that of coral communities, reflecting a greater evenness (Tukey test, *p* < 0.05).

Moreover, the 16S rRNA gene sequencing approach and statistical analyses revealed that the examined biotopes displayed different prokaryotic taxonomic profiles from one another at the phylum, class, and ASV levels (File S1, Supplementary Results and Figs. S2 and S3). *S. spinosulus* and *E. labiata* harboured prokaryotic communities that were distinct from those of their environmental vicinities (seawater and sediments), but also significantly different from each other in terms of structure and taxonomic composition (Figs. S2 and S3). Although all biotopes were dominated by the phyla *Pseudomonadota* and *Bacteroidota*, the relative abundance of these phyla changed considerably across biotopes, with the former displaying a pronounced dominance in octocorals and the latter showing higher relative abundances in seawater and sediments than in sponges and octocorals (File S1, Fig. S2). The same trend was observed for the two most dominant *Pseudomonadota* classes, *Alphaproteobacteria* and *Gammaproteobacteria*, which presented distinct relative abundances across all biotopes, with the former class being more abundant in octocorals and the latter displaying higher relative abundances in octocorals and seawater than in sponges and sediments (File S1, Fig. S3).

At the ASV level, the prokaryotic communities of the four biotopes were clearly unique and contrasting, thus providing support to the original motivation of this study. Indeed, a Principal Coordinates Analysis (PCoA) performed on the ASV profiles of the environmental prokaryotic communities revealed a sharp separation of the four biotopes (marine sponge, octocoral, seawater and sediment; *p* = 0.001, pairwise adonis < 0.05) (Fig. S2). Among the 20 most differentiating ASVs (according to the SIMPER test), those classified as *Endozoicomonas* (ASV23), *Aquimarina* (ASV22), besides two ASVs belonging to the Class *Alphaproteobacteria* (ASV46 and 235) and one ASV belonging to the family *Arenicellaceae* (Class *Gammaproteobacteria*, ASV128) – were associated with the *E. labiata* samples. Other ASVs such as *Constrictibacter* (ASV164), Subgroup 10 (*Acidobacteriota, Thermoanaerobaculaceae*) (ASV62 and 48) and ASVs only classified at high taxonomic ranks such as Anck6 (ASV146), *Dadabacteriales* (ASV78), *Chloroflexi* (ASV172), *Rhodothermaceae* (ASV90), *Sphingomonadales* (ASV120), subgroup 9 (*Acidobacteriota*, *Vicinamibacteria*) (ASV117) and *Nitrosopumilaceae* (ASV114) were associated with *S.* s*pinosulus*. ASV39, affiliated with *Nitrosopumilaceae* was located between the octocoral and the marine sponge samples, congruent with its presence in both host-associated biotopes while not being detected in the surrounding environments (Table S3). Moreover, a *Nitrosopumilus* phylotype (ASV56) was shared between the seawater and the marine sponge samples. Some other ASVs classified as Clade Ia (*Alphaproteobacteria*) (ASV44) and *Synechoccocus* CC9902 (ASV34 and 32) were located between the seawater and the octocoral and marine sponge samples, being indeed present in these three biotopes. None of the 20 most differentiating ASVs clustered close to the sediment group, although some of the abovementioned ASVs (ASV56, 44, 34 and 32) occurred in the sediment samples as well (Table S3).

Table S3 provides the abundance distributions of all ASVs (*n* = 5,673) detected across all environmental and enrichment culture samples analyzed in this study (*n* = 60).

### Artificial selection experiments

#### Enrichment cultures develop differentially according to their biotope of origin

**Fig. 2:**
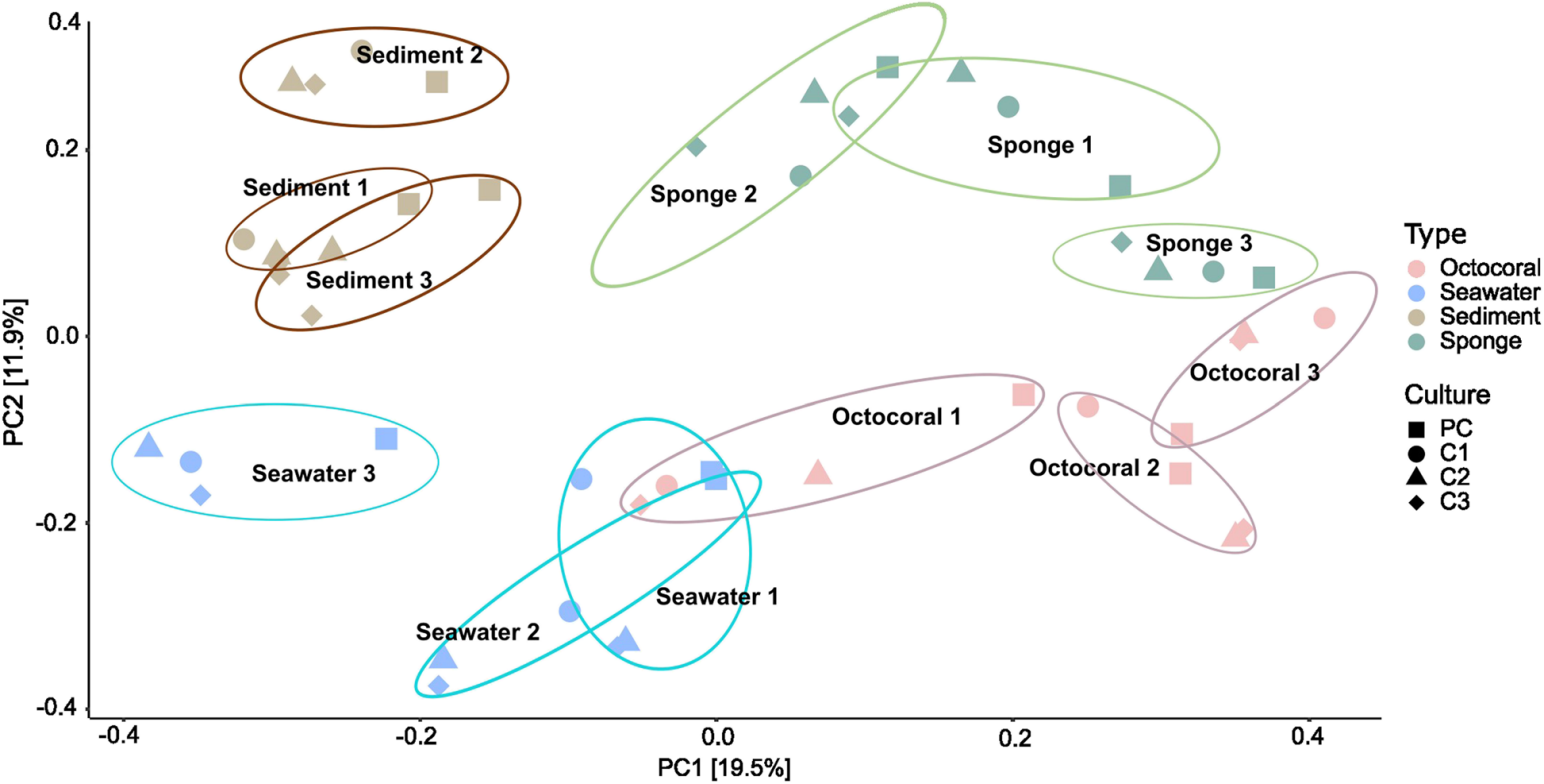
PCoA of prokaryotic communities from enrichment cultures performed on a Bray–Curtis distance matrix after Hellinger transformation of the ASV relative abundances. The different colors represent the biotope from which the prokaryotic communities were obtained. PC = preculture, C1 = enrichment culture 1, C2 = enrichment culture 2 and C3 = enrichment culture 3 during the artificial selection experiment. The ellipses were drawn manually

Multivariate analysis of all enrichment cultures showed a clear separation of the cultures according to their source biotope (Fig. 2). Moreover, within each biotope, we observed that enrichment cultures also formed distinguishable clusters according to the replicate experiment (Fig. 2). The separation of all enrichment cultures according to the source biotope (marine sponge, octocoral, seawater, and sediment) was statistically confirmed by a Welch MANOVA test (*p*-value = 0.001; pairwise adonis *p*-value = 0.006 for all pairs of biotopes). Furthermore, separate clustering of enrichment cultures (PC, C1, C2 and C3) derived from the same biotope but from different replicate experiments was confirmed by a PERMANOVA test (*p*-value = 0.001; pairwise adonis *p*-value = [0.018–0.042] for each pair of replicates). Moreover, the dispersion among the enrichment cultures PC, C1, C2 and C3 was higher than in their respective environmental samples (see PERMDISP values; Table S4) with one exception (for octocorals, PCs were less dispersed than the environmental samples).

#### Assessments of chitin polymer molecular weight suggest efficient chitin degradation in some cultures

**Fig. 3:**
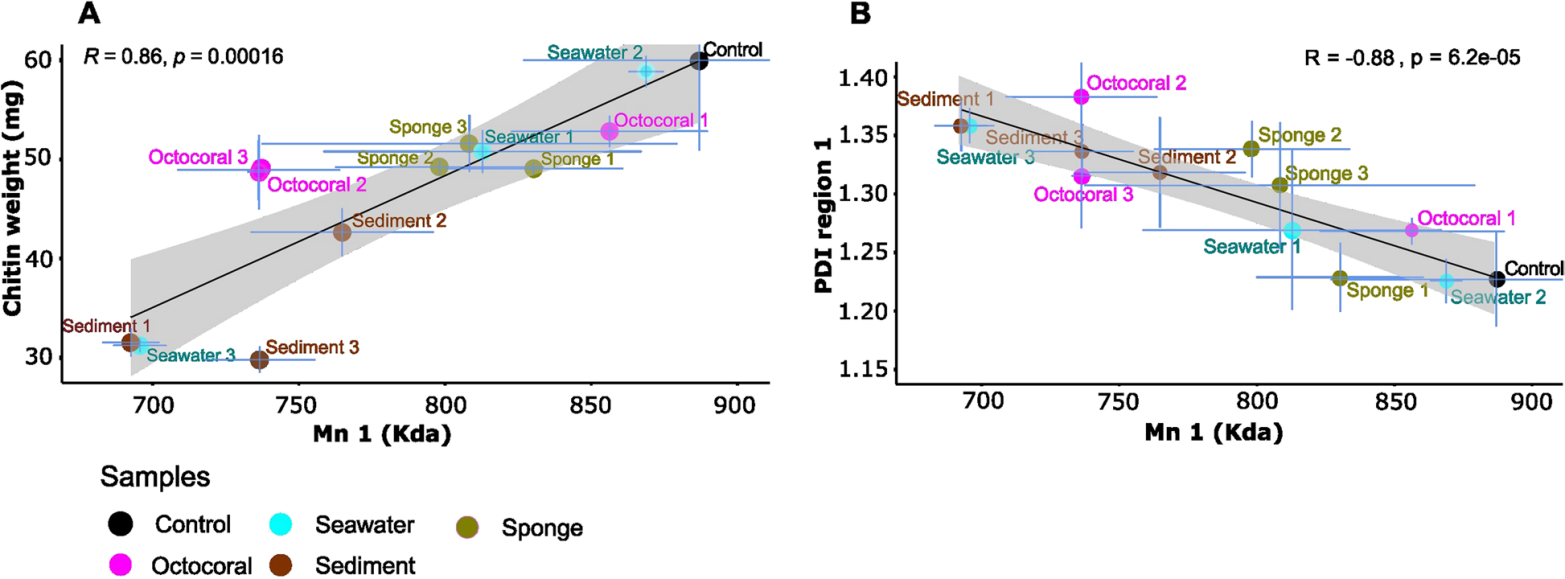
Correlations between parameters of chitin degradation in the enrichment culture C2 of all experiments. **A** Correlation between the numbered Average molecular weight of the first region (70.5—1,020 KDa)(Mn_1_) of the SEC chromatogram and the chitin weight (mg); **B** Correlation between Mn_1_ and polydispersity of the first region of the SEC chromatogram (PDI_1_). R and *p* values refer respectively to Pearson’s correlation coefficient and associated *p*-value. The light grey zone indicates a 95% confidence interval. Examples of chromatograms are depicted in Fig. S1

Using SEC, we could tap into the process of chitin degradation in the enrichment cultures. Indeed, a decrease in the polymer size (Mn_1_) indicates chitin degradation. Moreover, an increase in size variation (PDI_1_) suggests that the chitin polymer has been degraded in a range of diverse sizes. The Mn_1_ values of all C2 enrichment cultures, corresponding to the average size of the chitin polymer in these samples, were lower than the Mn_1_ value of the negative control, which consisted of chitin medium alone and was estimated at 887 KDa (Fig. 3; Tables S2, S5). This indicates that chitin degradation occurred in all enrichment cultures. However, we observed that estimated Mn_1_ values ranged from 693 KDa in sample SD1 to 869 KDa in sample SW2, being, overall, positively correlated with chitin weight measures (the lower the Mn_1_ estimate, the lower the weight of the remaining chitin) and negatively correlated with polydispersity (the lower the Mn_1_ estimate, the higher the polydispersity) (Fig. 3A and B, Table S5). In summary, the communities that exhibited low chitin weight usually broke down large chitin polymers into smaller molecules of a more diverse size range, resulting in a decrease in average size (Mn_1_) and an increase in size variation (PDI_1_) of chitin.

The trends above prompted us to establish a semi-quantitative rank of “chitin degradation efficiencies” based on the integration of chitin weight measures and Mn_1_ estimates (Table 1, see Table S2 for details). According to this scheme, we classified enrichment cultures SD1, SD3 and SW3 as “highly efficient” at degrading chitin, enrichment culture SD2 as “good”, cultures SP1-SP3, OC1-OC3, and SW1 as “moderate”, and culture SW2 as “low efficient” (Table 1). It is important to note that the dynamics of chitin degradation across all enrichment cultures could not be fully depicted using one single estimate or index alone. For instance, despite the correlations noted above, enrichment cultures OC2 and OC3 presented low Mn_1_ estimates, equivalent to that of culture SD3 (a “highly efficient” chitin degrading one) while presenting chitin weight loss of c. 25%, in the range of several “moderately efficient” consortia, being thus classified as “moderate” according to our conservative scheme (Table 1). This exemplifies that chitin weight measures alone, for instance, do not fully portray the chitin degradation dynamics in the samples. Altogether, sediment enrichment cultures were the most efficient and consistent at degrading chitin, followed by sponge and octocoral enrichment cultures (consistent “moderate” efficiencies) while seawater-derived cultures exhibited large variation in chitin degradation efficiencies.

**Table 1:** Simplified categorization of enrichment cultures into chitin degradation efficiencies based on chitin weight loss

Chitin degradation efficiency	Enrichment cultures	Weight loss (%)	Mn1 range (Da)
High	SW3, SD1, SD3	> 45%	6.93—7.37 + 05
Good	SD2	25—45%	7.65 + 05
Moderate	SP1, SP2, SP3, SW1, OC1, OC2, OC3	10—25%	7.36^a^—8.56 + 05
Low	SW2	< 10%	8.69 + 05

^a^low Mn_1_ values, suggesting high chitin degradation efficiency, were estimated for octocoral samples 2 and 3, which presented, however, moderate estimates of chitin weight loss according to the chitin weight measurement methodology employed in this study

#### Sharp taxonomy shifts between environmental samples and enrichment cultures were marked by the selection of potentially novel chitin-degrading taxa

**Fig. 4:**
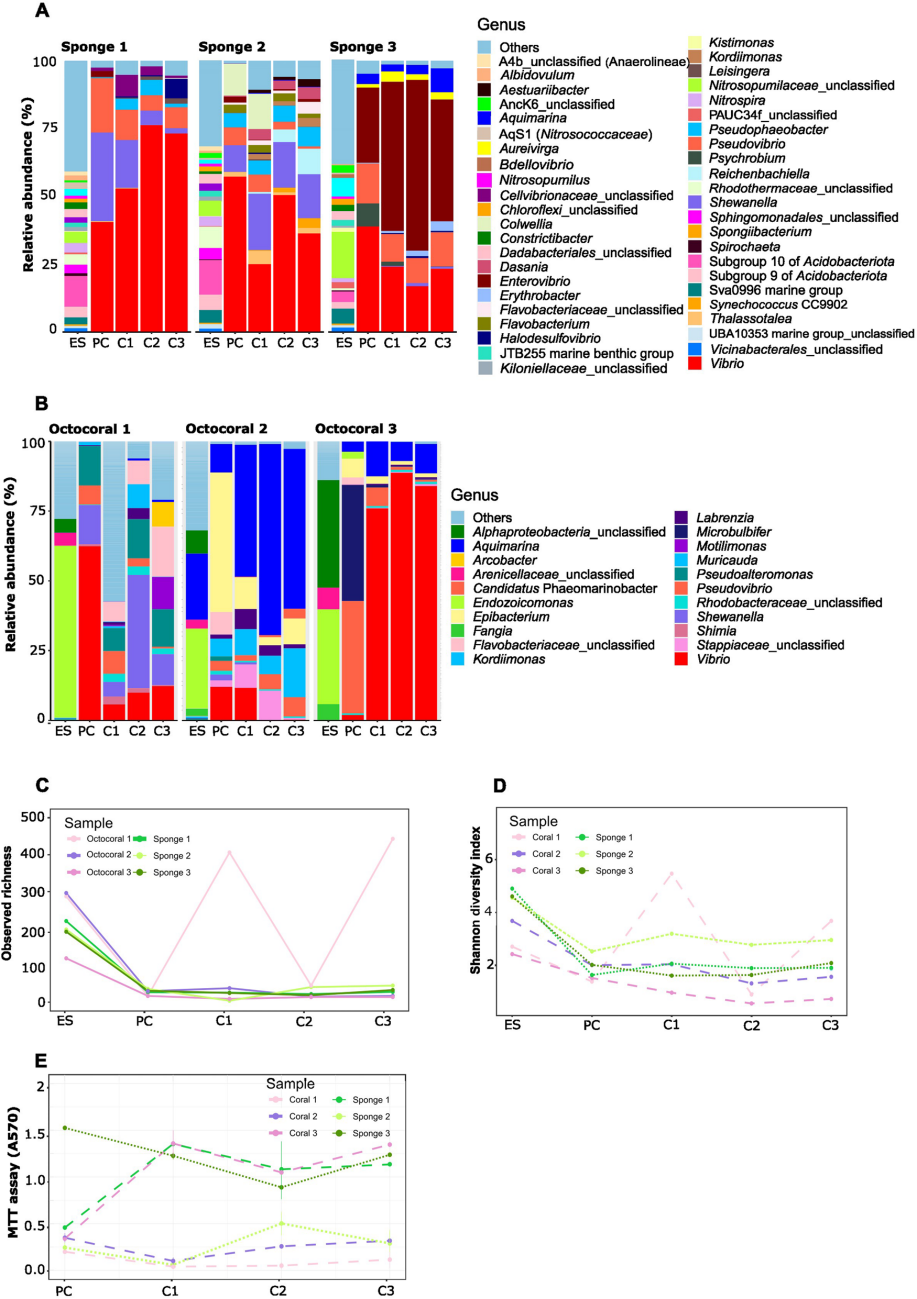
Taxonomic composition, alpha diversity and respiratory activity indicators of prokaryotic communities from marine sponges and octocorals during the artificial selection experiments. **A** and **B** Genus-level taxonomic composition of prokaryotic communities in environmental marine sponge and octocoral samples (ES) and their corresponding enrichment cultures (PC, C1, C2, and C3). In each of the six sub-datasets (one for each experiment), genera whose relative abundance was below 0.03% were merged into the category “Others”. **C** Observed ASV richness and **D** corresponding Shannon diversity index of prokaryotic communities from environmental samples and their corresponding enrichment cultures. **E)** Microbial activity assessments based on MTT assay results (Absorbance at 570 nm). Absorbance values of negative controls were subtracted from the values shown on the Y-axis

**Fig. 5:**
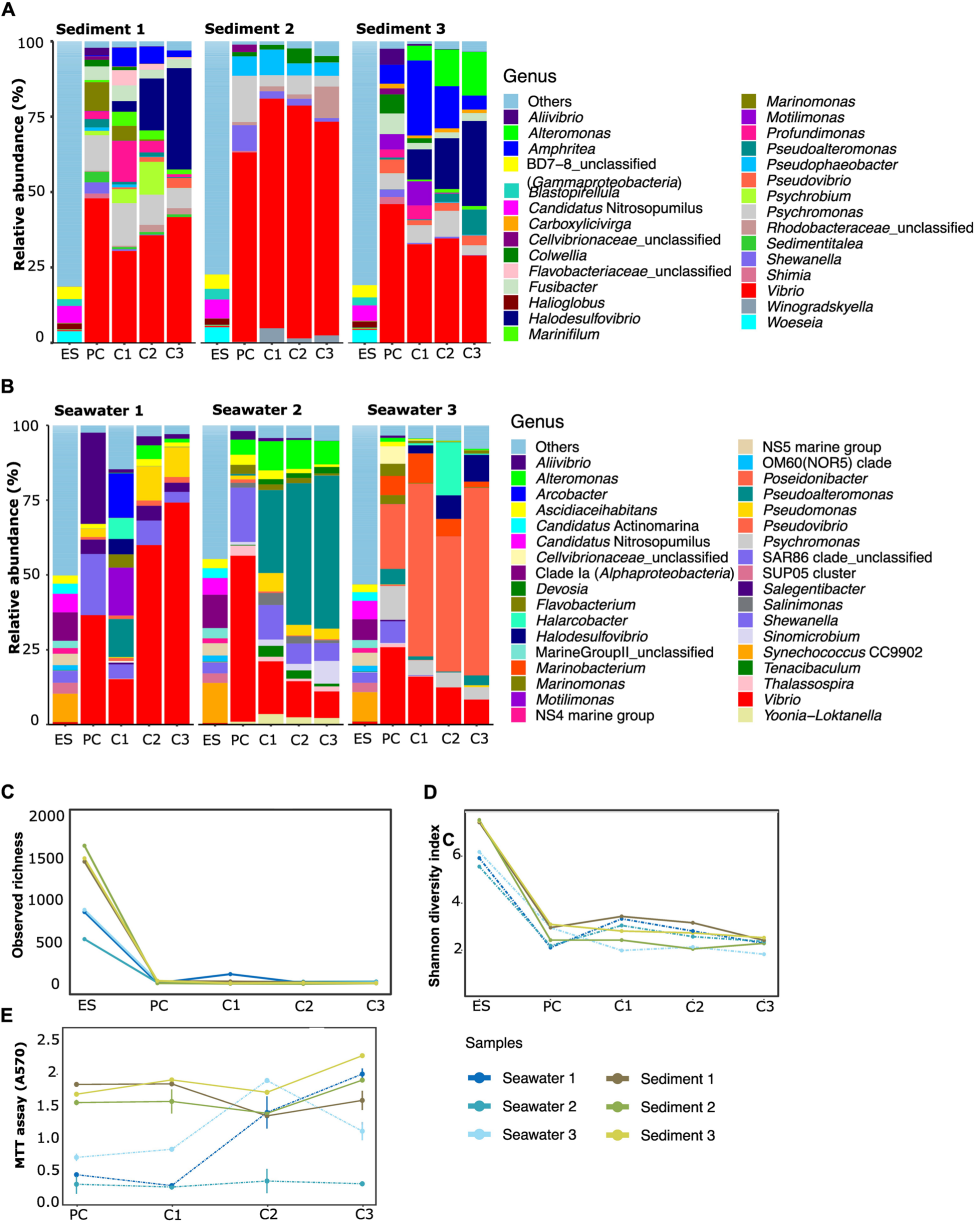
Taxonomic composition, alpha diversity, and bioactivity indicators of prokaryotic communities from seawater and sediments during the artificial selection experiments. **A** and **B** Genus-level taxonomic composition of prokaryotic communities in environmental sediment and seawater samples (ES) and their corresponding enrichment cultures (PC, C1, C2, and C3). In each of the six sub-datasets (one for each experiment), genera whose relative abundance was below 0.03% were merged into the category “Others”. **C** observed ASV richness and **D** corresponding Shannon diversity index of prokaryotic communities from environmental samples and their corresponding enrichment cultures. **E** Microbial activity assessments based on MTT assay results (Absorbance at 570 nm). Absorbance values of negative controls were subtracted from the values shown on the Y-axis

Figures 4 and 5 display genus-level taxonomic composition, ASV richness and diversity, and activity indicators of enrichment cultures derived from host-associated (Fig. 4A-E) and free-living (Fig. 5, A-E) biotopes. The repeated measure ANOVA tests performed on each biotope revealed significant differences in alpha-diversity measures, marked by a decrease in observed ASV richness and Shannon diversity indices, between the environmental samples and their corresponding enrichment cultures (*p*-value = [7.44 e-10—0.000122] for all samples from every biotope except for one octocoral sample (replicate #1). Post-hoc Tukey tests revealed significant differences in alpha-diversity only between the environmental samples and the PC, C1, C2 and C3 (*p*-value = [0.00293–3.44e-11]), again for every sample within each biotope except for octocoral replicate sample #1. In contrast, ASV richness and Shannon index did not change significantly further on in the selection process for each biotope, neither between PC and C1, nor between C1 and C2 or between C2 and C3 cultures (Tukey tests *p*-values = [0.396–1] for observed ASV richness and Tukey tests *p*-values = [0.479–1] for Shannon index). Furthermore, many of the dominant taxa in the environmental samples were not abundant (< 0.03%) or even undetectable in the enrichment cultures (Figs. 4A, B; and 5A, B). Conversely, dominant taxa in the enrichment cultures were poorly represented (< 0.1%) or absent in their corresponding environmental samples (Figs. 4A, B; and 5A, B; Table S6).

Respiratory activity (low to high) was recorded for all samples (Figs. 4E and 5E, Table S2). *Vibrio* ASV1 was consistently enriched in the cultures from all biotopes (Table S3). *Psychromonas* ASV26 was enriched in all cultures for which chitin degradation efficiency was classified as “good” or “high” (SD1, SD2, SD3, SW3) (Fig. 5A, Table S3), and a negative correlation was observed between the relative abundance of ASV26 and remaining chitin weight (Fig. S4). In all sediment cultures, *Vibrio* ASV13 was enriched. The enrichment cultures from sediment samples SD1 and SD3 were further dominated by *Halodesulfovibrio* ASV6, *Amphritea* ASV43, *Profundimonas* ASV60, *Fusibacter* ASV113 (in SD1) and ASV140 (in SD3), and *Pseudovibrio* ASV9. Unclassified *Rhodobacteraceae* (*Alphaproteobacteria*) ASV91 and *Pseudophaeobacter* ASV27 were also enriched in SD2 enrichment cultures; *Alteromonas* ASV29 was enriched in SD3- and *Psychrobium* ASV72 in SD1- enrichment cultures (Fig. 5A, Table S3).

Regarding the enrichment cultures from seawater SW3, perceived as highly efficient at degrading chitin according to our classification scheme (Table 1), C1, C2 and C3 were strongly dominated by *Poseidonibacter* ASV11. Of note, ASV11 was not enriched in the cultures from the two other seawater samples (SW1 and SW2, Fig. 5B) where chitin degradation was perceived as “moderate” or “low” (Table S2). Other dominant taxa in SW3 enrichment cultures were *Vibrio* ASV17, *Halodesulfovibrio* ASV6 and *Marinobacterium* ASV65. *Halarcobacter* ASV76 was abundant in the enrichment culture C2 of SW3. In SW1- and SW2- enrichment cultures, other taxa were enriched such as *Pseudomonas* ASV64, *Shewanella* ASV14 and *Alteromonas* ASV29. In the SW1 final enrichment culture, *Vibrio* ASV40 was also highly enriched and in SW2 enrichment cultures, *Pseudoalteromonas* ASV10 was highly enriched (Fig. 5B). All enriched communities from the marine sponge samples were dominated by *Pseudophaeobacter* ASV27, *Vibrio* ASV1 (a dominant ASV in sediment and seawater cultures as well) and ASV13, and by *Pseudovibrio* ASV9 (Fig. 4A, Table S3). Enrichment cultures of marine sponge samples SP1 and SP2 were also dominated by *Shewanella* ASV45 (in SP1) and ASV15 (in SP2) (Fig. 4A). SP2-derived cultures showed high abundance of *Vibrio* ASV17, and SP3-derived cultures of *Enterovibrio* ASV16 (Fig. 4A). Finally, the octocoral-derived enrichment cultures were largely dominated by *Aquimarina* ASV12 (sample OC2) and by *Vibrio* ASV1 (sample OC3), while OC1-enriched cultures were co-dominated by many taxa such as *Shewanella* ASV14, *Pseudoalteromonas* ASV10, *Flavobacteriaceae* ASV21 and *Motilimonas* ASV3 (Fig. 4B).

### Genome prospection reveals putative novel chitin degraders and consumers in artificially selected consortia

We performed a thorough analysis of chitin degradation and chitin-derivative utilization features across 102 publicly available bacterial genomes belonging to the genera *Aureivirga*, *Epibacterium*, *Halodesulfovibrio*, *Muricauda*, *Motilimonas*, *Pseudophaeobacter*, *Poseidonibacter*, *Psychromonas*, *Reichenbachiella* and *Thalassotalea* (Tables 2 and S7A,B). These genera were targeted because they were detected as dominant taxa in the experiments in which chitin was efficiently degraded (Fig. 3) whereas, to the best of our knowledge, current evidence for their roles as chitin degraders or consumers is either non-existent or scarce. Sequences coding for endochitinase domains were detected in the genome of all *Motilimonas, Reichenbachiella* and *Aureivirga* strains surveyed, and some *Halodesulfovibrio*, *Psychromonas, Muricauda,* and *Thalassotalea* strains (for details, see Table S7B). Exochitinase protein domains were detected in the genome of all *Motilimonas* strains and some *Reichenbachiella, Epibacterium*, *Psychromonas*, *Muricauda* and *Thalassotalea* strains*.* Sequences coding for polysaccharide deacetylase and N-acetylglucosamine utilization domains were detected in the genomes of the great majority of strains belonging to the different genera. Finally, sequences coding for chitin-binding protein domains were detected in the genomes of all *Motilimonas* strains and of some *Halodesulfovibrio* and *Psychromonas* strains. Compared to the other genomes, those of *Motilimonas* presented a considerably higher number of targeted sequences: 17 to 24 sequences per genome coding for domains of endochitinases, 3 to 4 for exochitinases, 5 to 11 for CBP and 3 to 4 for polysaccharide deacetylases (Table 2). *Motilimonas* was the single genus for which a positive score was recorded for all the searched functions across all genomes surveyed (Table S7B). Interestingly, genomes in the genera *Pseudophaeobacter* (found to be enriched in samples SD2 and SP2) and *Poseidonibacter* (sharply enriched in sample SW3, classified as “highly efficient” for chitin degradation) did not possess any endochitinase nor exochitinase coding sequences, but possessed polysaccharide deacetylation and N-acetyl-glucosamine utilization features. Based on these analyses, we proposed putative roles in chitin/chitin-derivative degradation and utilization for the ten bacterial genera examined more thoroughly in this study (Table 2).

**Table 2:** Pfam-based annotation overview of protein domains involved in chitin degradation found in publicly available genomes of putative novel chitin-degrading/-utilizing genera identified in this study^a^

Class	Genus	# genomes	Endo chitinases	Exo chitinases	CBP	Polysaccharide deacetylases	N-acetyl glucosamine	Suggested role/coding potential
*Gammaproteobacteria*	*Motilimonas*	4	4 [17–24]	4 [3, 4]	4 [5–11]	4 [3, 4]	4[1]	Chitin degrader via hydrolysis and deacetylation; COSs degrader via hydrolysis, GlcNAc consumer
*Alphaproteobacteria*	*Pseudophaeobacter*	4	0	0	0	4 [2–4]	4 [1, 2]	Chitin degrader via deacetylation, GlcNAc consumer
*Bacteroidia*	*Reichenbachiella*	5	5 [2, 3]	4 [0–5]	0	5 [2–4]	4 [0–3]	Chitin degrader via hydrolysis and deacetylation; putative COSs degrader via hydrolysis (strain-dependent feature), putative GlcNAc consumer (strain-dependent feature)
*Desulfovibrionia*	*Halodesulfovibrio*	5	2 [0–1]	2 [0–1]	2 [0–2]	5 [1, 2]	5 [1]	Putative chitin degrader via hydrolysis (strain-dependent feature); chitin degrader via deacetylation; GlcNAc consumer
*Bacteroidia*	*Aureivirga*	2	2 [2–4]	0	0	2 [1]	0	Chitin degrader via hydrolysis and deacetylation
*Alphaproteobacteria*	*Epibacterium*	4	0	2 [0–2]	0	4 [4]	4 [1]	Chitin degrader via deacetylation; putative COSs consumer (strain-dependent feature); GlcNAc consumer
*Gammaproteobacteria*	*Psychromonas*	18	13 [0–18]	5 [0–10]	5 [0–3]	18 [1–4]	16 [0–2]	Putative chitin degrader via hydrolysis (strain-dependent feature); chitin degrader via deacetylation; putative COSs degrader via hydrolysis (strain-dependent feature); putative GlcNAc consumer (strain-dependent feature)
*Bacteroidia*	*Muricauda*	26	6 [0–4]	25 [0–13]	0	14 [0–2]	26 [2–6]	Putative chitin degrader via hydrolysis (strain-dependent feature); chitin degrader via deacetylation; putative COSs degrader via hydrolysis (strain-dependent feature); GlcNAc consumer
*Campylobacteria*	*Poseidonibacter*	8	0	0	0	8 [1–3]	6 [0–1]	Chitin degrader via deacetylation; putative GlcNAc consumer (strain-dependent feature)
*Alphaproteobacteria*	*Thalassotalea*	26	8 [0–3]	19 [0–14]	0	26 [1, 2]	26 [3–9]	Putative chitin degrader via hydrolysis (strain-dependent feature); chitin degrader via deacetylation; putative COSs degrader via hydrolysis (strain-dependent feature); GlcNAc consumer

^a^Pfam annotations were performed on the IMG/M v.7 system using all genomes available for the examined taxa. Values in cells show the number of genomes within each genus for which a positive score was recorded for the screened function. Values in brackets display the range of variation in the number of protein domains found on the genomes of each genus which scored positive for that function. Pfam categories used to screen for chitin degradation-utilization features across genomes were as follows. Endochitinases: PF00182 (chitinase class I, GH19), PF00704 (GH18), PF08329 (chitinase A N-terminal domain), and PF06483 (chitinase C). Exochitinases: PF03174 and PF13290 (Chitobiase/beta-hexosaminidase C-terminal domains), PF03173 (Chitobiase/beta-hexosaminidase N-terminal domain), PF02838 (GH20, domain 2), PF00728 (GH20, catalytic domain), and PF14845 (beta-acetyl hexosaminidase like). Polysaccharide deacetylases: PF01522 (Polysaccharide deacetylase), PF04748 (divergent polysaccharide deacetylase)]. Chitin-binding proteins (CBP): PF01607 (Chitin binding Peritrophin-A domain, family 14), PF02839 (Carbohydrate-binding module family 5/12). N-acetylglucosamine utilization: PF01182 (Glucosamine-6-phosphate isomerases/6-phosphogluconolactonase)]. Table 2 synthesises Pfam annotation results obtained for 102 genomes across the ten genera examined (for details, see Tables S7A,B). Table S7A presents the full taxonomic string of each genus

## Discussion

This study employs artificial selection to enrich chitin-degrading microbial consortia from contrasting marine biotopes (sponges, octocorals, sediments and seawater). We highlight the recruitment of potential chitin degraders and utilizers from the original communities, placing a focus on the coding potential of enriched bacterial genera so far not described to play fundamental roles in the degradation of chitin and its derivatives. A detailed discussion on the structure of natural prokaryotic communities associated with each biotope is provided in Supplementary File S1.

### Chitin-based artificial selection applied to prokaryotic communities from marine biotopes leads to distinct chitin degrading consortia

This study demonstrates that applying a strictly equal artificial selection procedure to prokaryotic communities from four marine biotopes leads to different chitin degrading consortia. The natural prokaryotic communities of each biotope were clearly distinct from one another, which is line with earlier microbiome surveys of *S. spinosulus* and *E. labiata* specimens from the North Atlantic [25, 37] (for details, see Supplementary File S1). Moreover, artificial selection was found, in this study, to increase the natural variation among replicate samples (from the same biotope) observed in the environment. This can be explained by the likely combination of two factors: (1) natural differences that we observed in prokaryotic community assembly (*i.e.*, initial relative abundances of taxa in the biological replicates) driving distinct enrichment trajectories, and (2) sample processing procedures (*e.g.*, transportation, handling, glycerol preservation of the communities) which could alter the taxonomic composition of the inocula used for the selection experiments. This picture aligns with the concept of priority effects on microbiome assembly [38], whereby the final structure of a given community may rely on the order/timing of arrival of their founding members, which may be considerably influenced by stochastic events yet simultaneously dictate the successional changes underlying the assembly process. Priority effects have been evoked to explain sample-to-sample variability in algal [39] and fish larvae microbiomes [40, 41] and are considered a relevant phenomenon in the assembly of host-associated and free-living microbial communities across marine, freshwater and terrestrial ecosystems [38]. These outcomes revealed that each experiment was unique to some extent, even though deterministic factors (such as the ability to degrade chitin or utilizing COSs) simultaneously shaped the assembly of the enrichment cultures based on their source biotope. Moreover, although the same processing protocol was applied to all samples from the same biotope, slight variations in cell recovery and cell preservation efficiency (in glycerol stocks) might have as well contributed to the differences among enrichment cultures of the same origin observed in this study. To reduce variability among replicate enrichments from the same biotope, future studies may attempt pooling several subsamples (e.g., multiple excisions from the same sponge or coral specimen) to form a composite, representative sample of each replicate. Altogether, our data suggest that applying artificial selection to a range of distinct marine biotopes was successful and can increase the discoverability of novel chitin-degrading taxa and consortia which can be further studied by means of both phenotyping and genotyping.

### Artificial selection recruits low-abundance chitin degraders and utilizers

The artificial selection procedure employed in this study promotes low-abundance chitin degraders and utilizers within each biotope. Indeed, it was a consistent pattern that dominant taxa in the enrichment cultures were poorly represented or even not detectable in their corresponding environmental samples with the sequence depth employed. This suggests that the culturable bacteria from the marine sponge, octocoral, seawater, and sediment biotopes used in this study likely belong to the microbial rare biosphere within their natural environment. Microbial cultivation bias is a well-known phenomenon previously shown by several studies of free-living [42–44] and host-associated bacterial communities [45–47]. The high diversity of low abundance taxa present in natural microbial communities represents a vast collection of genetic features that contribute to a broad range of both established and potentially undiscovered microbial functions [48, 49]. It is also well established that low abundance, “rare” taxa can grow abundant under certain culture conditions [50] and degrade diverse pollutants, aromatic hydrocarbons [51–53], as well as complex polymers such as chitin as observed in this study. Enrichment of low abundance, chitin degrading microorganisms might as well occur in the natural environment under certain circumstances. Indeed, it has been recently shown that species in the known chitin degrading genus *Aquimarina* [5] usually belong to the rare biosphere of distinct marine biotopes [54] and may increase in abundance under certain conditions (e.g., in necrotic octocoral tissue [6, 55] and on the carapace of injured lobsters [56]).

Hardoim and colleagues suggested that the culturable fraction of bacterial symbionts of the marine sponges *Sarcotragus spinosulus* and *Ircinia variabilis* consisted primarily of low abundance species [37]. Conversely, dominant symbionts belonging to the *Rhodothermales* order and the *Endozoicomonas* genus were recently suggested, through genome-resolved metagenomics studies, to play a key role in chitin degradation in marine sponges [12] and octocorals [6], respectively. However, although these taxa were abundant in the here examined *S. spinosulus* (*Rhodothermaceae* ASV90; Fig. 3) and *E. labiata* (*Endozoicomonas* ASV23; Fig. 3) specimens, respectively, they escaped enrichment via artificial selection as attempted in this study. This is likely due to the sampling, transportation and cultivation procedures employed for artificial selection (including the retrieval of microbial cell homogenates from the samples, their conservation, the culture medium, and incubation conditions). Therefore, alternative techniques to recover these symbionts in culture need to be implemented to harness their metabolism. These include varying culture conditions (adjusting pH, temperature, and physical–chemical conditions), modifying the composition of the medium (for instance, by incorporating host chemical cues, other forms of chitin or chitin derived from different host animals), or adopting gentler sampling processing methods. Another future step could be to express the chitin-degrading enzymes of these symbionts, such as chitinases, without the need for cultivation. This can be achieved through targeted gene cloning and expression for chitinase genes [57, 58] or directly by chitinase gene synthesis based on their metagenomic sequences and subsequent cloning and expression.

### Consortia from host-associated and free-living communities show different chitin degradation efficiencies

We found that chitin degradation efficiency varied across enrichment cultures, both within and across biotopes, as indicated by chitin weight loss and Mn1 estimates. This outcome may be explained by several factors. First, sediment and seawater harbor greater microbial diversity than host-associated environments, as shown in this study and previous research [59, 60]. A higher diversity increases the likelihood of selecting microbes that thrive under specific enrichment conditions. Second, differences in chitin degradation potential have been reported for the different biotopes. Indeed, in a previous study [5], seawater, sediment, marine sponge, and octocoral communities from the same location exhibited distinct chitin degradation gene distributions. Seawater and sediment metagenomes contained significantly higher proportions of endochitinase genes than those of marine sponges, which might explain why our consortia enriched from the former biotopes exhibited higher chitin degradation efficiency. Additionally, sediments and seawater metagenomes harbored significantly more exochitinase and N-acetylglucosamine utilization genes than those of octocorals. No major differences in deacetylase gene abundances were detected between the metagenomes of the different biotopes. Thus, it was hypothesized that free-living and host-associated bacterial communities could primarily degrade chitin via different mechanisms, with hydrolysis and deacetylation being, eventually, favoured in the former and latter communities. However, chitin deacetylase activity is less detectable by SEC and by measuring the chitin weight loss, as it mainly modifies the chemical structure (removing acetyl groups) without significantly altering polymer size [61]. Regarding the only experiment in which chitin degradation efficiency was low (SW2), although speculative, it is possible that fewer active cells from the glycerol stock were introduced into the culture, affecting chitin degradation dynamics.

### Cross-feeding may promote co-existence of chitin degraders and utilizers in enrichment cultures

Many well-known chitin degraders were enriched in the cultures derived from host-associated consortia, such as *Vibrio* [5, 62], *Shewanella* [5, 63], *Pseudoalteromonas* [5, 64], *Pseudomonas* [65, 66], *Aquimarina* [5, 67], *Alteromonas* [68], and *Enterovibrio* [5]. In the enrichment cultures derived from sediment and seawater bacterial communities, some of the enriched taxa were also already known to be chitin degraders such as *Vibrio* [5, 62], *Alteromonas* [68] and *Pseudoalteromonas* [5, 64]. Some others are known to be chitin utilizers such as representatives of the *Rhodobacteraceae* (*Alphaproteobacteria*) family [5, 6] and of the genus *Pseudovibrio* in the *Stappiaceae* family [5]. This suggests the co-existence of chitin degraders and utilizers through hypothesized cross-feeding mechanisms. Indeed, chitin degraders might make excess chitin degradation products available (COSs, chitosan, GlcNAc) which are used by other chitinolytic bacteria (i.e., chitin utilizers). This process, although difficult to demonstrate experimentally, was already suggested in previous studies [5, 6, 9, 69–71]. Moreover, to the best of our knowledge, many other genera enriched in cultures from all biotopes are so far unknown or understudied for their role as chitin degraders and/or utilizers. Among them, the roles of ten representative genera were inferred in this study based on functional annotation of several dozens of genomes publicly available. We suggest that *Motilimonas, Reichenbachiella, Halodesulfovibrio, Aureivirga* and *Psychromonas* are potential chitin degraders by means of hydrolysis*. Epibacterium* may have a role in chitin utilization/COSs degradation via hydrolysis. *Pseudophaeobacter* and *Poseidonibacter* may have a role in chitin degradation via deacetylation and in GlcNAc consumption. Moreover, all genera examined, except *Aureivirga*, are classified as potential GlcNAc utilisers. Wright and colleagues [14] applied an artificial selection process on microbial communities from bulk marine debris (Devon, UK) using varying incubation times (e.g., 4 days and 9 days) over several transfers, observing a few enriched taxa in common with our study. These include well-known chitin degraders such as *Vibrio*, *Alteromonas* and *Pseudoalteromonas* and other taxa less known for their role in chitin degradation such as *Muricauda* and *Thalassotalea*. Genomes available for these two taxa were found, in our study, to possess endochitinase genes, suggesting a potential role for these organisms as chitin degraders by means of hydrolysis in multiple biotopes. Performing artificial selection on several marine biotopes may thus increase the chance of obtaining different taxa involved in chitin degradation. Moreover, Wright and colleagues [14] suggested that successive transfers over short time periods (e.g., 4 days) favours the selection of chitin-degrading bacteria in the *Gammaproteobacteria* class while reducing the abundance of chitin utilizers/“grazers” of COSs, such as representative members of the *Alphaproteobacteria* class. The seven-day incubation period employed in our study led to the promotion of relatively stable communities most likely composed of a mix of chitin degraders and utilizers over the course of the experiment (29 days from pre-culture to culture C3). Taken together, our results suggest that a versatile chitin catabolism, involving the breakdown of chitin and COSs via endo and exochitinase-mediated hydrolysis, deacetylation into chitosan, and N-acetylglucosamine utilization features, was assembled in enrichment cultures via artificial selection from multiple marine biotopes. This indicates that substrate cross-feeding among bacteria is a possible mechanism maintaining the diversity of chitin degraders and chitin-derivative utilizers in the enrichment cultures, as observed experimentally by Pontrelli and colleagues for specific combinations of marine bacteria [71].

## Conclusion

In this study, distinct bacterial consortia efficient at degrading chitin composed by known chitin degraders, known chitin utilizers and many taxa not yet known or only poorly studied for their role(s) in chitin degradation were selected from several marine biotopes. The latter taxa (e.g., *Motilimonas*, *Muricauda*, *Halodesulfovibrio*, *Psychromonas*, *Reichenbachiella,* among others) are potential key players in marine chitin degradation. Future research may employ top-down methods to isolate strains from the enriched communities, studying the chitin degradation abilities of individual strains through commercially available chitinase assays and/or chitin degradation activity screenings on colloidal chitin agar plates, and reconstructing even simpler communities. In addition, bottom-up techniques may include diluting the communities to specifically select the most active chitin degraders and utilizers. Recently, DNA-Stable Isotope Probing (DNA-SIP) has been implemented to monitor the incorporation of ^13^C labelled chitin by natural microbial communities [72]. Such an approach holds promise in unveiling chitin degraders, utilizers and scavengers (those not directly acting on chitin or COSs but feeding on metabolic by-products of chitin degraders and utilizers – see e.g., [71]) in future artificial selection experiments, possibly strengthening cross-feeding hypotheses often raised to explain the co-existence of chitin-transforming microorganisms in natural and artificial settings.

As a future perspective, isolating the dominant taxa from enriched cultures and characterizing their chitin degradation capacities through functional assays and genome sequencing will provide deeper insights into their metabolic potential. Additionally, metagenomic sequencing of chitin-degrading consortia represents a powerful approach to elucidate the ecological roles of individual taxa and to uncover novel chitinolytic enzymes from marine environments.

## Supplementary Information

Supplementary Material 1.

Supplementary Material 2.

## Data Availability

The amplicon data (Table S8) are available in the Sequence Read Archives (SRA) under the project accession number PRJNA999598, sample accession numbers SAMN36737297 to SAMN36737311, SAMN36737334 to SAMN36737348, SAMN36743901 to SAMN367433915 and SAMN36744262 to SAMN36744262 to SAMN36744276; run accession numbers from SRR25451171 to SRR25451185, SRR25451141 to SRR25451155, SRR25451652 to SRR25451666 and SRR25451336 to SRR25451350.

## References

[1] Gooday GW. The Ecology of Chitin Degradation. In: Marshall KC, editor. Advances in Microbial Ecology. Boston, MA: Springer, US; 1990. p. 387–430.

[2] Johnston J. Conditions of life in the sea: a short account of quantitative marine biological research. University Press. 1908.

[3] Heidelberg JF, Heidelberg KB, Colwell RR. Bacteria of the γ-subclass *Proteobacteria* associated with zooplankton in Chesapeake Bay. Appl Environ Microbiol. 2002. 10.1128/AEM.68.11.5498-5507.2002. 12406743 10.1128/AEM.68.11.5498-5507.2002 PMC129896

[4] Poulicek M, Jeuniaux C. Chitin Biomass in marine sediments. In: Chitin and Chitosan (G. Skjak-Brack, T. Anthonsen, P. Sandford, ed.), Elsevier Applied Science. 1989;1:151–5.

[5] Raimundo I, Silva R, Meunier L, Valente SM, Lago-Lestón A, Keller-Costa T, et al. Functional metagenomics reveals differential chitin degradation and utilization features across free-living and host-associated marine microbiomes. Microbiome. 2021;9:43. 33583433 10.1186/s40168-020-00970-2 PMC7883442

[6] Keller-Costa T, Kozma L, Silva SG, Toscan R, Gonçalves J, Lago-Lestón A, et al. Metagenomics-resolved genomics provides novel insights into chitin turnover, metabolic specialization, and niche partitioning in the octocoral microbiome. Microbiome. 2022;10:151. 36138466 10.1186/s40168-022-01343-7 PMC9502895

[7] Herwig RP, Pellerin NB, Irgens RL, Maki JS, Staley JT. Chitinolytic bacteria and chitin mineralization in the marine waters and sediments along the Antarctic Peninsula. FEMS Microbiol Lett. 1988;53:101–11.

[8] Souza CP, Almeida BC, Colwell RR, Rivera ING. The importance of chitin in the marine environment. Mar Biotechnol. 2011;13:823–30. 10.1007/s10126-011-9388-1 21607543

[9] Beier S, Bertilsson S. Bacterial chitin degradation—mechanisms and ecophysiological strategies. Front Microbiol. 2013. 10.3389/fmicb.2013.00149. 23785358 10.3389/fmicb.2013.00149 PMC3682446

[10] Hobel CFV, Marteinsson VT, Hreggvidsson GÓ, Kristjánsson JK. Investigation of the microbial ecology of intertidal hot springs by using diversity analysis of 16S rRNA and chitinase genes. Appl Environ Microbiol. 2005;71:2771–6. 15870372 10.1128/AEM.71.5.2771-2776.2005 PMC1087530

[11] Cretoiu MS, Kielak AM, Al-Soud WA, Sørensen SJ, van Elsas JD. Mining of unexplored habitats for novel chitinases—chiA as a helper gene proxy in metagenomics. Appl Microbiol Biotechnol. 2012;94:1347–58. 22526805 10.1007/s00253-012-4057-5 PMC3353111

[12] Silva R. Uncovering the chitin degradation potential of the microbiomes of marine sponges and octocorals (unpublished doctoral dissertation). Universidade de Lisboa, Instituto Superior Técnico, Lisbon. Universidade de Lisboa, Instituto Superior Técnico; 2023.

[13] Souza CP, Burbano-Rosero EM, Almeida BC, Martins GG, Albertini LS, Rivera ING. Culture medium for isolating chitinolytic bacteria from seawater and plankton. World J Microbiol Biotechnol. 2009;25:2079–82.

[14] Wright RJ, Gibson MI, Christie-Oleza JA. Understanding microbial community dynamics to improve optimal microbiome selection. Microbiome. 2019;7:85. 31159875 10.1186/s40168-019-0702-x PMC6547603

[15] Ascon-Cabrera M, Lebeault J-M. Selection of xenobiotic-degrading microorganisms in a biphasic aqueous-organic system. Appl Environ Microbiol. 1993;59:1717–24. 16348949 10.1128/aem.59.6.1717-1724.1993 PMC182150

[16] Swenson W, Arendt J, Wilson DS. Artificial selection of microbial ecosystems for 3-chloroaniline biodegradation. Environ Microbiol. 2000;2:564–71. 11233164 10.1046/j.1462-2920.2000.00140.x

[17] Borchert E, Hammerschmidt K, Hentschel U, Deines P. Enhancing microbial pollutant degradation by integrating eco-evolutionary principles with environmental biotechnology. Trends Microbiol. 2021;29:908–18. 33812769 10.1016/j.tim.2021.03.002

[18] Saima, Kuddus M, Roohi, Ahmad IZ. Isolation of novel chitinolytic bacteria and production optimization of extracellular chitinase. Journal of Genetic Engineering and Biotechnology. 2013;11:39–46.

[19] Morin-Crini N, Lichtfouse E, Torri G, Crini G. Applications of chitosan in food, pharmaceuticals, medicine, cosmetics, agriculture, textiles, pulp and paper, biotechnology, and environmental chemistry. Environ Chem Lett. 2019;17:1667–92.

[20] Paulsen SS, Andersen B, Gram L, Machado H. Biological potential of chitinolytic marine bacteria. Mar Drugs. 2016;14: 230. 27999269 10.3390/md14120230 PMC5192467

[21] Abdulkarim A, Isa MT, Abdulsalam S, Muhammad AJ, Ameh AO. Extraction and characterisation of chitin and chitosan from mussel shell. Civ Environ Res. 2013;3:108–14.

[22] Itoh T, Hibi T, Fujii Y, Sugimoto I, Fujiwara A, Suzuki F, et al. Cooperative degradation of chitin by extracellular and cell surface-expressed chitinases from *Paenibacillus* sp. strain FPU-7. Appl Environ Microbiol. 2013;79:7482–90. 24077704 10.1128/AEM.02483-13 PMC3837743

[23] Hamed I, Özogul F, Regenstein JM. Industrial applications of crustacean by-products (chitin, chitosan, and chitooligosaccharides): a review. Trends Food Sci Technol. 2016;48:40–50.

[24] El Knidri H, Belaabed R, Addaou A, Laajeb A, Lahsini A. Extraction, chemical modification and characterization of chitin and chitosan. Int J Biol Macromol. 2018;120:1181–9. 30172808 10.1016/j.ijbiomac.2018.08.139

[25] Keller-Costa T, Eriksson D, Gonçalves JMS, Gomes NCM, Lago-Lestón A, Costa R. The gorgonian coral *Eunicella labiata* hosts a distinct prokaryotic consortium amenable to cultivation. FEMS Microbiol Ecol. 2017;93: fix143. 10.1093/femsec/fix143 29069352

[26] Shrivastava A. Polymerization. In: Introduction to Plastics Engineering. Elsevier; 2018. p. 17–48.

[27] Apprill A, McNally S, Parsons R, Weber L. Minor revision to V4 region SSU rRNA 806R gene primer greatly increases detection of SAR11 bacterioplankton. Aquat Microb Ecol. 2015;75:129–37.

[28] Parada AE, Needham DM, Fuhrman JA. Every base matters: assessing small subunit rRNA primers for marine microbiomes with mock communities, time series and global field samples. Environ Microbiol. 2016;18:1403–14. 26271760 10.1111/1462-2920.13023

[29] Callahan BJ, McMurdie PJ, Rosen MJ, Han AW, Johnson AJA, Holmes SP. DADA2: High-resolution sample inference from Illumina amplicon data. Nat Methods. 2016;13:581–3. 27214047 10.1038/nmeth.3869 PMC4927377

[30] Mukherjee S, Huntemann M, Ivanova N, Kyrpides NC, Pati A. Large-scale contamination of microbial isolate genomes by Illumina PhiX control. Stand in Genomic Sci. 2015;10:18. 26203331 10.1186/1944-3277-10-18 PMC4511556

[31] Quast C, Pruesse E, Yilmaz P, Gerken J, Schweer T, Yarza P, et al. The SILVA ribosomal RNA gene database project: improved data processing and web-based tools. Nucleic Acids Res. 2012;41:D590–6. 23193283 10.1093/nar/gks1219 PMC3531112

[32] Yilmaz P, Parfrey LW, Yarza P, Gerken J, Pruesse E, Quast C, et al. The SILVA and “All-species Living Tree Project (LTP)” taxonomic frameworks. Nucleic Acids Res. 2014;42:D643–8. 24293649 10.1093/nar/gkt1209 PMC3965112

[33] McMurdie PJ, Holmes S. phyloseq: an R package for reproducible interactive analysis and graphics of microbiome census data. PLoS One. 2013;8:1–11. 10.1371/journal.pone.0061217 PMC3632530 23630581

[34] Wickham H, François R, Henry L, Müller K, Vaughan D, Software P, et al. dplyr: A Grammar of Data Manipulation. 2023. https://CRAN.R-project.org/package=dplyr. Accessed 15 Apr 2023.

[35] Wickham H. ggplot2: Elegant Graphics for Data Analysis. Springer International Publishing. 2016.

[36] Chen I-MA, Chu K, Palaniappan K, Pillay M, Ratner A, Huang J, et al. IMG/M v.5.0: an integrated data management and comparative analysis system for microbial genomes and microbiomes. Nucleic Acids Res. 2019;47:D666–77. 10.1093/nar/gky901 PMC6323987 30289528

[37] Hardoim CCP, Cardinale M, Cùcio ACB, Esteves AIS, Berg G, Xavier JR, et al. Effects of sample handling and cultivation bias on the specificity of bacterial communities in keratose marine sponges. Front Microbiol. 2014;5:611. 10.3389/fmicb.2014.00611. 10.3389/fmicb.2014.00611 PMC4235377 25477868

[38] Debray R, Herbert RA, Jaffe AL, Crits-Christoph A, Power ME, Koskella B. Priority effects in microbiome assembly. Nat Rev Microbiol. 2022;20:109–21. 34453137 10.1038/s41579-021-00604-w

[39] Burke C, Thomas T, Lewis M, Steinberg P, Kjelleberg S. Composition, uniqueness and variability of the epiphytic bacterial community of the green alga *Ulva australis*. ISME J. 2011;5:590–600. 21048801 10.1038/ismej.2010.164 PMC3105733

[40] Califano G, Castanho S, Soares F, Ribeiro L, Cox CJ, Mata L, et al. Molecular Taxonomic Profiling of Bacterial Communities in a Gilthead Seabream (Sparus aurata) Hatchery. Front Microbiol. 2017;8:204. 10.3389/fmicb.2017.00204. 10.3389/fmicb.2017.00204 PMC5306143 28261166

[41] Sanches-Fernandes GMM, Califano G, Castanho S, Soares F, Ribeiro L, Pousão-Ferreira P, et al. Effects of live feed manipulation with algal-derived antimicrobial metabolites on fish larvae microbiome assembly: a molecular-based assessment. Aquac Res. 2022;53:1062–83.

[42] Kogure K, Shimizu U, Taga N. A tentative direct microscopic method for counting living marine bacteria. Can J Microbiol. 1979;26:318–23. 10.1139/m79-063 378340

[43] Staley JT, Konopka A. Measurement of in situ activities of nonphotosynthetic microorganisms in aquatic and terrestrial habitats. Annu Rev Microbiol. 1985;39:321–46. 3904603 10.1146/annurev.mi.39.100185.001541

[44] Amann RI, Ludwig W, Schleifer KH. Phylogenetic identification and in situ detection of individual microbial cells without cultivation. Microbiol Rev. 1995;59:143–69. 7535888 10.1128/mr.59.1.143-169.1995 PMC239358

[45] Friedrich AB, Fischer I, Proksch P, Hacker J, Hentschel U. Temporal variation of the microbial community associated with the Mediterranean sponge *Aplysina aerophoba*. FEMS Microbiol Ecol. 2001;38:105–13.

[46] Webster NS, Hill RT. The culturable microbial community of the Great Barrier Reef sponge Rhopaloeides odorabile is dominated by an alpha-proteobacterium. 2001;138:843–51.

[47] Taylor MW, Tsai P, Simister RL, Deines P, Botte E, Ericson G, et al. ‘Sponge-specific’ bacteria are widespread (but rare) in diverse marine environments. ISME J. 2013;7:438–43. 23038173 10.1038/ismej.2012.111 PMC3554410

[48] Elshahed MS, Youssef NH, Spain AM, Sheik C, Najar FZ, Sukharnikov LO, et al. Novelty and uniqueness patterns of rare members of the soil biosphere. Appl Environ Microbiol. 2008;74:5422–8. 18606799 10.1128/AEM.00410-08 PMC2546616

[49] Lynch MDJ, Neufeld JD. Ecology and exploration of the rare biosphere. Nat Rev Microbiol. 2015;13:217–29. 25730701 10.1038/nrmicro3400

[50] Shade A, Jones SE, Caporaso JG, Handelsman J, Knight R, Fierer N, et al. Conditionally rare taxa disproportionately contribute to temporal changes in microbial diversity. mBio. 2014. 10.1128/mbio.01371-14. 25028427 10.1128/mBio.01371-14 PMC4161262

[51] Sauret C, Séverin T, Vétion G, Guigue C, Goutx M, Pujo-Pay M, et al. ‘Rare biosphere’ bacteria as key phenanthrene degraders in coastal seawaters. Environ Pollut. 2014;194:246–53. 25156140 10.1016/j.envpol.2014.07.024

[52] Fuentes S, Barra B, Caporaso JG, Seeger M. From rare to dominant: a fine-tuned soil bacterial bloom during petroleum hydrocarbon bioremediation. Appl Environ Microbiol. 2016;82:888–96. 26590285 10.1128/AEM.02625-15 PMC4725283

[53] Wang Y, Hatt JK, Tsementzi D, Rodriguez-R LM, Ruiz-Pérez CA, Weigand MR, et al. Quantifying the importance of the rare biosphere for microbial community response to organic pollutants in a freshwater ecosystem. Appl Environ Microbiol. 2017;83:e03321-e3416. 28258138 10.1128/AEM.03321-16 PMC5377499

[54] Silva SG, Paula P, Da Silva JP, Mil-Homens D, Teixeira MC, Fialho AM, et al. Insights into the antimicrobial activities and metabolomes of *Aquimarina* (*Flavobacteriaceae, Bacteroidetes*) species from the rare marine biosphere. Mar Drugs. 2022;20: 423. 35877716 10.3390/md20070423 PMC9323603

[55] Keller-Costa T. Metagenomic insights into the taxonomy, function, and dysbiosis of prokaryotic communities in octocorals. Microbiome. 2021;9:72. 10.1186/s40168-021-01031-y. 10.1186/s40168-021-01031-y PMC7993494 33766108

[56] Ooi MC, Goulden EF, Trotter AJ, Smith GG, Bridle AR. *Aquimarina* sp. associated with a cuticular disease of cultured larval palinurid and scyllarid lobsters. Front Microbiol. 2020;11:573588. 33162955 10.3389/fmicb.2020.573588 PMC7581904

[57] Gan Z, Yang J, Tao N, Yu Z, Zhang K-Q. Cloning and expression analysis of a chitinase gene *Crchi1* from the mycoparasitic fungus *Clonostachys rosea* (syn *Gliocladium roseum*). J Microbiol (Seoul, Korea). 2007;45:422–30. 17978802

[58] Wang S, Fang X, Liang K, Li S, Han S, Zhu T. Cloning, expression and antifungal effect of the recombinant chitinase from *Streptomyces sampsonii* KJ40. Cienc Rural. 2023;53:e20210663.

[59] Cleary DFR, Swierts T, Coelho FJRC, Polónia ARM, Huang YM, Ferreira MRS, et al. The sponge microbiome within the greater coral reef microbial metacommunity. Nat Commun. 2019;10:1644. 30967538 10.1038/s41467-019-09537-8 PMC6456735

[60] Hoshino T, Doi H, Uramoto G-I, Wörmer L, Adhikari RR, Xiao N, et al. Global diversity of microbial communities in marine sediment. Proc Natl Acad Sci USA. 2020;117:27587–97. 33077589 10.1073/pnas.1919139117 PMC7959581

[61] Zhao Y, Park R-D, Muzzarelli RAA. Chitin deacetylases: properties and applications. Mar Drugs. 2010;8:24–46. 20161969 10.3390/md8010024 PMC2817921

[62] Svitil AL, Chadhain S, Moore JA, Kirchman DL. Chitin degradation proteins produced by the marine bacterium *Vibrio harveyi* growing on different forms of chitin. Appl Environ Microbiol. 1997;63:408–13. 16535505 10.1128/aem.63.2.408-413.1997 PMC1389511

[63] Laribi-Habchi H, Bouacem K, Allala F, Jabeur F, Selama O, Mechri S, et al. Characterization of chitinase from *Shewanella* inventionis HE3 with bio-insecticidal effect against granary weevil, *Sitophilus granarius Linnaeus* (*Coleoptera*: *Curculionidae*). Process Biochem. 2020;97:222–33.

[64] Techkarnjanaruk S, Goodman AE. Multiple genes involved in chitin degradation from the marine bacterium *Pseudoalteromonas* sp. strain S91. Microbiology. 1999;145:925–34. 10220172 10.1099/13500872-145-4-925

[65] Folders J, Algra J, Roelofs MS, van Loon LC, Tommassen J, Bitter W. Characterization of *Pseudomonas aeruginosa* chitinase, a gradually secreted protein. J Bacteriol. 2001;183:7044–52. 11717261 10.1128/JB.183.24.7044-7052.2001 PMC95551

[66] Chen L, Jiang H, Cheng Q, Chen J, Wu G, Kumar A, et al. Enhanced nematicidal potential of the chitinase pachi from *Pseudomonas aeruginosa* in association with Cry21Aa. Sci Rep. 2015;5:14395. 26400097 10.1038/srep14395 PMC4585872

[67] Nedashkovskaya O, Kim S-G, Stenkova A, Kukhlevskiy A, Zhukova N, Mikhailov V. *Aquimarina algiphila* sp. nov., a chitin degrading bacterium isolated from the red alga *Tichocarpus crinitus*. Int J Syst Evol Microbiol. 2018. 10.1099/ijsem.0.002606. 29458485 10.1099/ijsem.0.002606

[68] Tsujibo H, Fujimoto K, Kimura Y, Miyamoto K, Imada C, Okami Y, et al. Purification and characterization of beta-N-acetylglucosaminidase from Alteromonas sp strain O-7. Biosci Biotechnol Biochem. 1995;59:1135–6. 7613001 10.1271/bbb.59.1135

[69] Daniels M, Stubbusch AKM, Held NA, Schubert OT, Ackermann M. Effects of interspecies interactions on marine community ecosystem function. bioRxiv. 2022;08(26):505414. 10.1101/2022.08.26.505414.

[70] Daniels M, van Vliet S, Ackermann M. Changes in interactions over ecological time scales influence single-cell growth dynamics in a metabolically coupled marine microbial community. ISME J. 2023;17:406–16. 36611102 10.1038/s41396-022-01312-w PMC9938273

[71] Pontrelli S, Szabo R, Pollak S, Schwartzman J, Ledezma-Tejeida D, Cordero OX, et al. Metabolic cross-feeding structures the assembly of polysaccharide degrading communities. Sci Adv. 2022;8: eabk3076. 35196097 10.1126/sciadv.abk3076 PMC8865766

[72] Martinović T, Mašínová T, López-Mondéjar R, Jansa J, Štursová M, Starke R, et al. Microbial utilization of simple and complex carbon compounds in a temperate forest soil. Soil Biol Biochem. 2022;173: 108786.

