# Supplementary material for: An artificial selection procedure enriches for known and suspected chitin degraders from the prokaryotic rare biosphere of multiple marine biotopes
Source: BMC Microbiol. 2025 Nov 25;25:778. doi: 10.1186/s12866-025-04218-7 (PMC12648933; doi:10.1186/s12866-025-04218-7)
Supplement: Supplementary file 1 — Supplementary Material 1. [file 12866_2025_4218_MOESM1_ESM.docx]

**Supplementary File S1**

**An artificial selection procedure enriches for known and suspected chitin degraders from the prokaryotic rare biosphere of multiple marine biotopes**

Authors: Laurence Meunier^1^, Tina Keller-Costa^2,3^, David Cannella^4^, Jorge Gonçalves^5^, Etienne Dechamps^1^, Matilde Marques^2,3^, Rodrigo Costa^2,3*^† and #Isabelle F. George^1,6^†

Affiliations ^1^Laboratory of Ecology of Aquatic Systems, Brussels Bioengineering School, Université Libre de Bruxelles (ULB), Brussels, Belgium

^2^Institute for Bioengineering and Biosciences (iBB) and Institute for Health and Bioeconomy (i4HB), Instituto Superior Técnico (IST), Universidade de Lisboa, Lisbon, Portugal

^3^Department of Bioengineering, Instituto Superior Técnico (IST), Universidade de Lisboa, Lisbon, Portugal

^4^PhotoBioCatalysis Unit, Crop Nutrition and Biostimulation Lab (CPBL) and Biomass Transformation Lab (BTL), Brussels Bioengineering School, Université Libre de Bruxelles, Brussels, Belgium

^5^Algarve Centre of Marine Sciences (CCMAR), Universidade do Algarve (UALG), Faro, Portugal

^6^Laboratory of Marine Biology, Department of Biology, Université Libre de Bruxelles, Brussels, Belgium

† these corresponding authors contributed to this study equally.

* Correspondence: Rodrigo Costa, rodrigocosta@tecnico.ulisboa.pt, +351 21 841 7339 Isabelle F. George Isabelle.George@ulb.be ☏ +32 2 650 59 24,

Keywords: prokaryotic communities, enrichment cultures, chitinase, marine sponge, octocoral, size exclusion chromatography.

**Supplementary Methodology**

**SM.1. Preparation of microbial cell pellets**

Microbial cell pellets were obtained from marine sponge and octocoral tissue according to the method described by [1] with minor modifications. Pieces of marine sponge and octocoral tissue of each specimen (1 g each), prepared as described above, were grinded in 9 mL of sterile Calcium-Magnesium-free-ASW (CMFASW. Composition: 27 g L−1 NaCl, 1 g L−1 NaSO_4_, 0.8 g L−1 KCl and 0.18 g L−1 NaHCO_3_) using a sterile mortar and a pestle. The homogenates were then centrifuged at 4 °C at 500 g for 2 min to remove the host-derived tissue (pellet). Thereafter, the supernatant was centrifuged at 4 °C at 10,000 g for 15 min to recover the microbial cell pellet. To prepare microbial cell pellets from seawater samples, approximately 1 L of seawater was filtered through 0.22 µm pore size membranes (Millipore, MERCK). The seawater membranes were then cut into small pieces with a sterile scissor, mixed with a lab spoon of sterile 2-mm glass beads and with 50 mL of CMFASW. To prepare microbial cell pellets from sediment samples, approximately 1 g of sediment was mixed with a lab spoon of sterile 2 mm glass beads and with 9 mL of CMFASW. Afterwards, the cell suspensions from seawater and sediment were vortexed twice at max. speed for 30-60 sec with a 10 min interval to detach the microbial cells from the filters and sediment, respectively. All suspensions were then centrifuged at 4 ºC at 500 g for 2 min to decant glass beads plus seawater filter pieces or sediment particles. Then, the supernatant was centrifuged at 4 ºC at 10,000 g for 15 min to recover the microbial cell pellet. Each cell pellet (seawater, sediment, marine sponge, and octocoral) was resuspended in 830 µL of sterile ASW and transferred into sterile, 2 mL cryo-vials equipped with 150 µL of sterile 100% glycerol and 20 µL of pure, 100 % DMSO. These glycerol stocks were stored at -80 °C until further use.

**SM.2. MTT viability assays**

This assay was adapted from [2]. Briefly, 60 µL of MTT (tetrazolium salt; Sigma-Aldrich/Merck, Germany) were added to 200 µL of each enrichment culture (in technical triplicates) in sterile 96-well microplates. The microplates were incubated for 30 min at 37 °C. Then, plates were centrifuged at 10,000 g for 7 min and the supernatant was removed. The remaining, reduced formazan cell pellet was dissolved in 200 µL of pure DMSO, the microplates were centrifuged again at 10,000 g for 7 min, and the absorbance at 570 nm (A570) was measured on the supernatant. If the A570 value of a sample was higher than the A570 of the negative control (chitin-based enrichment culture medium incubated under the same conditions as the (pre)cultures but without bacteria), it was interpreted as an indication of bacterial growth.

**SM.3. Chitin degradation assessment**

To assess chitin degradation features across C2 enrichment cultures, chitin powder was recovered from 5 mL of the enrichment cultures C2 (in triplicate) and from 5 mL of the negative control C- (in triplicate) by centrifugation (5 min at 2000 rpm), followed by two washing steps of the pellet with MilliQ water. The pellet was subsequently dried in heating blocks at 70 °C (DRB200; Hach, USA) until reaching a constant weight. Then, the pellet was solubilized into chitosan. To deacetylate the chitin polymers, 1 mL of NaOH 50% was added to 10 mg of chitin (adapted from [3–5]). The reaction tubes were vortexed for 5 sec and then incubated at 60 °C for 1 day. Afterwards, the obtained chitosan was centrifuged (5 min at 7,000 rpm) and the chitosan pellet washed three times with MilliQ water.

SEC was performed at ~25°C using a combination of one pre-column PSS 10μ NOVEMA 3000 $Å$ (8 mm diameter x 50 mm length; Polymer Standards Service GmhH, Mainz, Germany) and three columns 10μ NOVEMA 3000 $Å$ NOVEMA MAX high (8 mm diameter x 300 mm length; Polymer Standards Service GmhH, Mainz, Germany) connected sequentially using an UHPLC system (Dionex Ultimate 3000, Sunnyvale) equipped with a refracting index sensor. The eluent, an aqueous solution of formic acid 0.03 % and NaNO_3_ 0.05 M, was pumped in isocratic mode at a flow rate of 1 mL/ min. Injected samples contained all 1.5 mg of chitosan (processed upstream from chitin) dissolved in 1 mL of eluent. Linear pullulan with a range from 0.180 to 1020 KDa (ReadyCal-Kit Pullulan high from Polymer Standards Service GmhH, Mainz, Germany; Fig. S2A) was used as standard to calculate the regression equation to convert the obtained retention time into molecular weight of the chitosan polymer (which resulted from the conversion of chitin).

The polymeric parameters were calculated from the signals detected in time slices within a significant region of the whole chromatogram, hereafter termed “region 1”, spanning 70.5 KDa – 1,020 KDa in molecular weight and thus excluding small sized oligomers (Fig. S1). For “region 1” of the chromatograms, the numbered average molecular weight Mn_1_ was calculated as follows:

$$Mn= \frac{(\sum Ni*Mi)}{\sum Ni}$$

Where *i* is a slice (1.666 x 10^-3^ min) of the region, *Mi* is the molecular weight and *Ni* is the intensity of the signal.

Mw, the weight average molecular weight, was calculated as follows:

$$Mw= \frac{(\sum Ni*{Mi}^{2})}{\sum Ni*Mi}$$

where *i* is a slice of the region, *Mi* is the molecular weight and *Ni* is the intensity of the signal.

Polydispersity PDI is a measure of the broadness of the peak and was calculated as follows:

$$PDI=\frac{Mn}{Mw}$$

Correlation analyses were thereafter performed to assess the strength of the relationship between chitin degradation parameters assessed in this study. Specifically, Pearson correlations were computed between the estimated Mn_1_ values obtained for all C2 enrichment cultures and the corresponding values recorded for weight of remaining chitin in the cultures and PDI_1_ using the *ggscatter* function (*cor.method*= “pearson”) from the ggpubr package (v 0.5.0;[6]) in R. These analyses allowed us to assess hypotheses of a positive correlation between Mn_1_ and remaining chitin weight values (the higher the chitin weight remaining in the tube, the higher the molecular mass of the chitin polymer) and a negative correlation between PDI_1_ and Mn_1_ values (the higher the polydispersity of the chitin peaks, the lower the estimates of chitin molecular weight, suggesting that the large chitin polymer is being broken down into smaller oligos of different sizes).

**SM.4. Processing of the 16S rRNA gene sequencing data**

First, the FilterandTrim function from DADA2 was used to remove the reads with Ns (maxN=0) using the following settings: trim the end of the forward and reverse reads at a specific base pair position where the quality of the majority of reads dropped under Q=30 (truncLen = c(240,150)), filter out the reads belonging to the PhiX bacteriophage (rm.phix = TRUE), select reads with high number of errors (maxEE = 2) and truncate those with high levels of error at the earliest occurrence of a quality score that is equal to or lower than 2 (truncQ=2; the value 2 is utilized by Illumina as a read end quality indicator). After combining all identical sequence reads into ‘‘unique sequences” (each associated with the number of reads of each sequence), the DADA2 algorithm inferred Amplicon Sequence Variants (ASVs). Paired-end sequences were merged (using the mergeSequenceTables() function from DADA2) and chimeric ASVs were removed (using the DADA2 function removeBimeraDenovo() function) from the ASVs table. Taxonomy (Kingdom, Phylum, Class, Order, Family and Genus) of each ASV was then assigned using the SILVA database version 138.1

**SM.5. Taxonomic composition**

The final dataset containing abundance distributions of prokaryotic ASVs across all samples was used to generate stacked bar charts in multiple combinations to facilitate data visualization. All barplots were generated using the R packages phyloseq (v1.38.0; [7], dplyr (v1.8.6,[8]) and ggplot2 (v 3.4.0; [9]).

**SM.6. Alpha diversity**

Alpha-diversity metrics were determined for prokaryotic communities of environmental samples and enrichment cultures using non-rarefied data, since rarefaction curves of all samples reached a plateau (*data not shown*), indicating that the prokaryotic diversity in each sample was exhausted with the sequencing depth employed. Observed prokaryotic richness (ASV counts) and diversity (Shannon-Wiener diversity index calculated from the abundance distributions of ASVs) were obtained for each sample using the *estimate_richness* function from the phyloseq package (v1.38.0; [7]).

For the prokaryotic communities of environmental samples, boxplots representing observed richness (ASV counts) and the Shannon index were plotted using the *qplot* function from the ggpubr package (v0.5.0; [6]). The normality and homoscedasticity of the data were confirmed using Shapiro-Wilk and Levene tests, respectively, included in the car package (v3.1-1) in R [10]. Then, an ANOVA test was performed to check whether differences in alpha diversity measures between groups of samples were significant. If the ANOVA test was significant, a post-hoc Tukey test was then carried out to test for significant differences between pairs of groups. A repeated measure ANOVA test was conducted using the *anova_test()* function from Rstatix package [11] to determine whether differences in observed richness and Shannon diversity index between prokaryotic communities from the environmental samples and from the corresponding enrichment cultures PC, C1, C2 and C3 were significant. The repeated measure ANOVA makes the following assumptions about the data: i) no significant outliers and ii) normality of the data. All functions used to test the assumptions belong to the Rstatix package [11]. If the test was significant, post-hoc pairwise Tukey tests were conducted to test for significant differences in alpha diversity measures between the environmental samples and the corresponding preculture samples using the *tukey_hsd()* function from Rstatix package [11] in R. All statistical tests were performed in R version 4.1.2.

**SM.7. Beta diversity analyses**

Two beta diversity analyses were performed in this study: i) one to determine whether differences in prokaryotic community structure occurred among the environmental samples of each biotope under study (seawater, sediment, marine sponge and octocoral) and ii) one to determine whether such differences occurred between the enrichment cultures derived from each biotope and from each biotope replicate. For each analysis, the ASV data were first Hellinger-transformed (square root of ASV relative abundances). Then, a Bray-Curtis similarity matrix was calculated using the phyloseq package from R (v1.38.0; [7]). A Principal Coordinates Analysis (PCoA) was generated for each analysis to ordinate the samples based on the Bray-Curtis matrix. Ordination diagrams were drawn using the ggplot2 package (v 3.4.0;[9]) in R.

To check for significant differences in prokaryotic community structure between environmental samples and/or enrichments cultures, a Permutational Analysis of Variance (PERMANOVA) [12] or a Welch MANOVA [13] was performed depending on the homogeneity of variance between groups of samples. First, a PERMDISP test was conducted to test for significant differences in dispersion between groups of samples using the *betadisp()* function from the vegan R package [12]. The PERMDISP test was also used to check whether the artificial selection procedure decreased the natural variation observed in the environment. If the PERMDISP test was non-significant, meaning that the dispersion between the groups of samples was similar, the PERMANOVA test was conducted, otherwise the Welch MANOVA test was conducted. The PERMANOVA test was performed on each Bray-Curtis distance matrix with 999 permutations in R using the *adonis2* function from the vegan R package (v2.6-4;[12]). The Welch MANOVA test was conducted using the *Wd Test* function from the *MicEco* package [14]. If the PERMANOVA/ Welch MANOVA test was significant, a Post Hoc test was performed using the function *pairwiseAdonis::pairwise.adonis* from the pairwiseAdonis R package (v0.4.1; [15]) to ascertain significant differences between pairs of groups.

For the analysis including only the environmental samples, ASVs that are the main contributors to community dissimilarities were identified with a Similarity Percentage (SIMPER) test on the Hellinger-transformed data using the PAST software (version 4.10;[16]). The 20 most differentiating ASVs were plotted in the PCoA graph.

**Supplementary Results**

**SR.1 Each biotope displays a different prokaryotic taxonomic profile at the phylum level**

Total community DNA concentrations (ng/µL) ranged from 18.8 – 41.7, 18 – 21.4, 6.8 – 9.9 and 1.4 – 6.2 among the sponge, octocoral, sediment and seawater environmental samples, respectively. The V4 region of the 16S rRNA gene was successfully amplified and sequenced from all samples, resulting in 1076734 reads and a total of 4727 ASVs analysed from the environmental samples.

Marine sponge, seawater and sediment samples were dominated by multiple phyla compared with the octocoral samples which were largely dominated by *Proteobacteria* (hereafter called *Pseudomonadota* to follow the current bacterial nomenclature) (Fig.S2A). Moreover, all samples had only a few phyla with a relative abundance below 1 % (c. 5 % when pooled) (Fig.S2A). Although *Pseudomonadota* was a dominant phylum in all biotopes, its relative abundance differed between samples (c. 25 % in *S. spinosulus*, 55-80 % in *E. labiata*, c. 30 % in sediment and c. 45 % in seawater samples) (Fig.S2A). All biotopes were co-dominated by *Bacteroidota* at different relative abundances (c. 7 % in *S. spinosulus*, c. 30 % in *E. labiata* sample 2 and c. 20 % in sediment and seawater samples). *S. spinosulus* and seawater samples also shared two other dominant phyla: *Actinobacteriota* (15-25 % in *S. spinosulus* and c. 6 % in seawater samples) and *Nitrososphaeorota* (formerly *Crenarchaeota*) (7-20 % in *S. spinosulus* and c. 7 % in seawater samples). Some other dominant phyla were specific to one biotope. In the *S. spinosulus* samples, further dominant phyla were *Acidobacteriota* (15-25 %), *Chloroflexi* (10-25 %) and *Gemmatimonadota* (c. 7 %). In *E. labiata,* sample 2 was well represented by *Planctomycetota* reads (c. 4 %) while *Spirochaetota* reads were more prevalent in samples 1 and 3 (c. 2 %). Finally, in all sediment samples, *Planctomycetota* (c. 10 %), *Desulfobacterota* (c. 7 %) and *Crenarchaeota* (c.7 %) were among the most dominant phyla while *Cyanobacteria* (c. 12 %) and *Thermoplasmatota* (c. 6 %) ranked among the dominant phyla in seawater.

At the class level, host-associated microbial communities were also very different from each other and from their surrounding environment (Fig. S3). Two classes were shared among the four biotopes but with different relative abundances. This was the case with *Gammaproteobacteria* (i.e., 45-75 % - *E. labiata*; 12-20 % - *S. spinosulus*; c. 30 % - sediment; c. 20 % seawater) and *Alphaproteobacteria* (i.e., 10-45 % - *E. labiata*; c. 5 % - *S. spinosulus*; c. 3 % - sediment; c. 20 % - seawater). Some other classes were shared between the marine sponge biotope and its surrounding biotopes such as *Nitrososphaeria* (c. 5-18 % - *S. spinosulus*; c. 6 % - sediment; c. 9 % - seawater) and *Acidimicrobiia* (c. 2 % in each biotope). Moreover, *Bacteroidia* was shared between *E.labiata* and its surrounding environment (1- 30 % - *E. labiata*; 10-15 % - seawater; c. 20 % -sediment). Finally, some classes were dominant only in one biotope such as *Spirochaetia* in *E.labiata*, samples 1 and 3 (c. 2 %), *Vicinamibacteria* (*Acidobacteria*) and *Thermoanaerobaculia (Acidobacteria)* (c. 7.5 % and 3-15 % respectively) in the *S.Spinosulus*, *Polyangia (Myxococcota)* in sediment (c. 2 %), and *Thermoplasta* and *Cyanobacteria* in seawater (c. 7 % and c. 15 % respectively).

**SR.2 *Sarcotragus spinosulus* (marine sponge) and *Eunicella labiata* (octocoral) host similar ASV richness but distinct ASV diversity**

Observed richness (ASV counts) and Shannon diversity indices (Fig. S2B) were significantly different across biotopes (ANOVA p-value < 0.05). However, prokaryotic communities from the marine sponge and octocoral samples assessed in this study had a similar ASV count (Tukey test, p-value > 0.05), even though their Shannon diversity indices were significantly different (Tukey test, p-value < 0.05) with higher values estimated for the marine sponge samples. Thus, the prokaryotic communities of the marine sponge samples have a greater evenness at the ASV level than those of the octocoral samples. The observed richness and Shannon index of the sediment samples were significantly higher than those of other biotopes (Tukey test, p-values < 0.05). Altogether, the host-associated prokaryotic communities were less rich and diverse than the ones of seawater and sediment (Tukey test, p-value < 0.05).

**Supplementary Discussion**

**SD.1 The marine sponge *Sarcotragus spinosulus* and the octocoral *Eunicella labiata* from the same vicinities select and maintain distinct prokaryotic communities**

This is the first study to directly compare the prokaryotic communities of the marine sponge *Sarcotragus spinosulus* and the octocoral *Eunicella labiata,* which are characteristic organisms of the Atlanto-Mediterranean benthic fauna (Fig. S2). We strengthen previous findings by Hardoim et al. [17] and Keller-Costa et al. [1] on the distinctiveness of the prokaryotic communities of the two host organisms in comparison with their surrounding environments (seawater and sediment). Moreover, the prokaryotic communities of the two host species were distinct from each other, with specific prokaryotic taxa associated with either animal even though samples were collected close to each other. The prokaryotic communities of *S.* *spinosulus* displayed higher ASV diversity indices than the ones of *E. labiata*, even though the ASV richness of both communities were similar. We also observed higher richness of bacterial classes and phyla in association with *S. spinosulus* than with *E. labiata*. Taken together, these outcomes highlight higher dominance of prokaryotic taxa in *E. labiata* than in *S. spinosulus*, in agreement with previous evidences that, in the octocoral microbiome, few taxa dominate the community [18, 19]. Indeed, *E. labiata* prokaryotic communities were largely dominated by *Pseudomonadota* while in *S. spinosulus* multiple phyla co-dominated. This is congruent with previous observations of the *E. labiata* [1] and *S. spinosulus* [17] microbiomes. However, while *Actinobacteria* and *Poribacteria* were among the dominant phyla in *S. spinosulus* (c. 20 and 11 % relative abundance, respectively) according to the study conducted by [17], their estimated relative abundances were much lower in our study (c. 3 and 1 %, respectively). While such contrasting results can to some extent be attributed to the different sampling years, seasons, and locations employed in both studies, primer choice is likely an important factor underlying the observed differences, as primers recommended in the Earth Microbiome Project currently in use have been noted to underrepresent poribacterial abundances in natural settings [20]. Nevertheless, congruent with the current literature, the prokaryotic phyla found in association with *S. spinosulus* in this study typically rank among the dominant members of the microbiome of high microbial abundance sponges. The archaeal genus *Nitrosopumilus,* which currently belongs to the new validated phylum *Nitrososphaerota* (formerly *Crenarchaeota*, [21]) was found in this study as the dominant archaeon in association with *S. spinosulus*, congruent with the findings of [17]. Species in the genus *Nitrosopumilus* such as *N. maritimus* can grow chemolithoautotrophically by performing the first step of the nitrification process, which consists in aerobically oxidizing ammonia to nitrite [22], thus likely contributing to ammonia removal and nitrogen cycling within the marine sponge holobiont [23]. Congruent with and extending the trends observed at phylum and class levels, this study uncovered numerous ASVs specific to, or sharply enriched in, the prokaryotic communities of *E. labiata* and *S. spinosulus* (for details, see Fig. 1C and Table S3).

**Supplementary Figures**


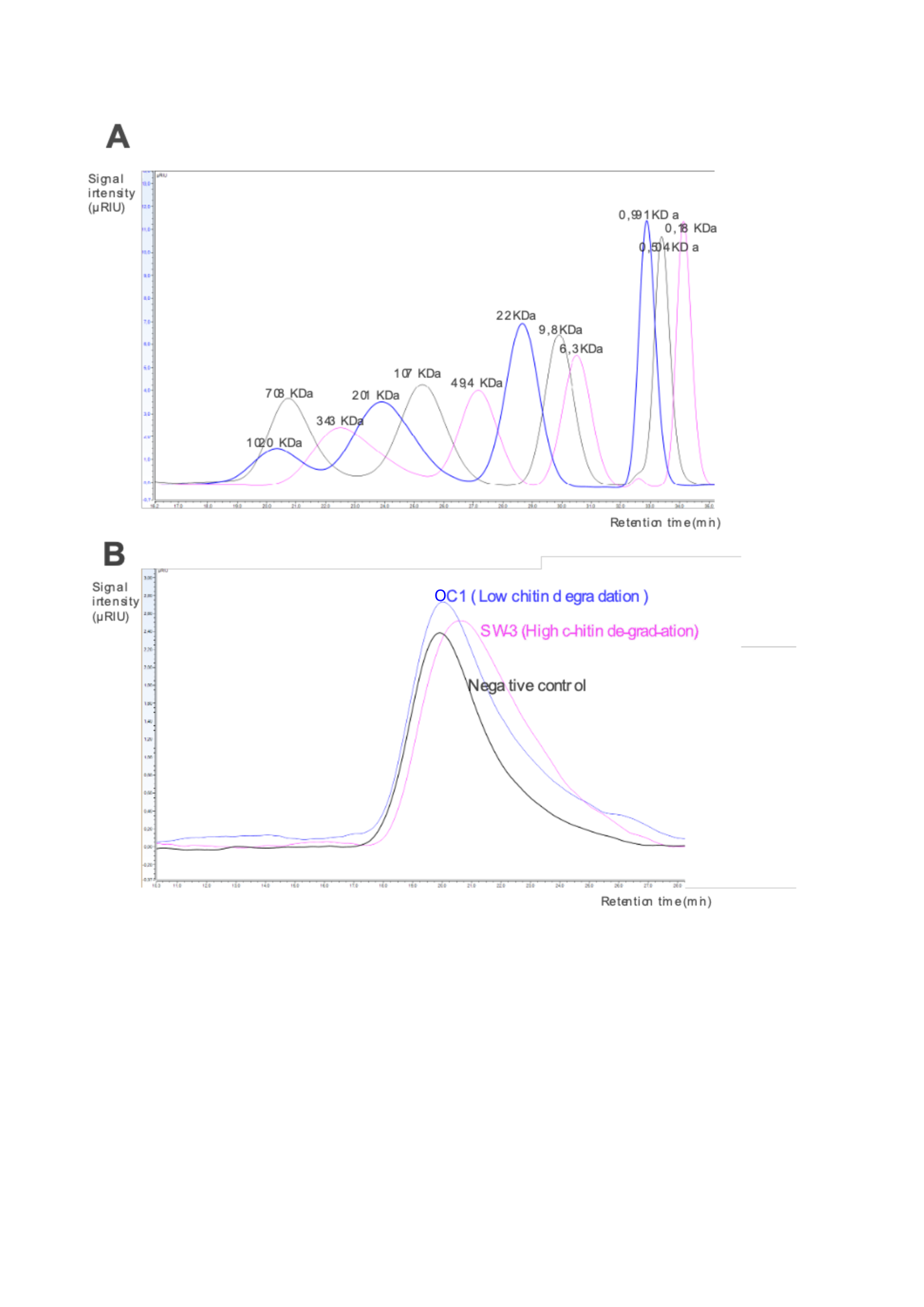


**Figure S1**: SEC chromatograms computed by Chromeleon software. A) represents the standards of linear pullulans with the molecular weight of each peak indicated graphically, those were used to calculate the molecular parameters of the unknown samples starting from the retention time and signal intensity, B) represents the main region of the obtained chitin chromatograms (including molecular weights from 80 KDa to >1.000 KDa), defined as region 1. We report 3 cases: a native undigested chitin or negative control (black line); a sample in which the chitin was extensively degraded (SW3 pink line; Mn_1_ decreased compared to Mn_1_ of the control); finally a sample in which chitin was marginally degraded (OC1; blue line; Mn_1_ is similar to Mn_1_ the control).


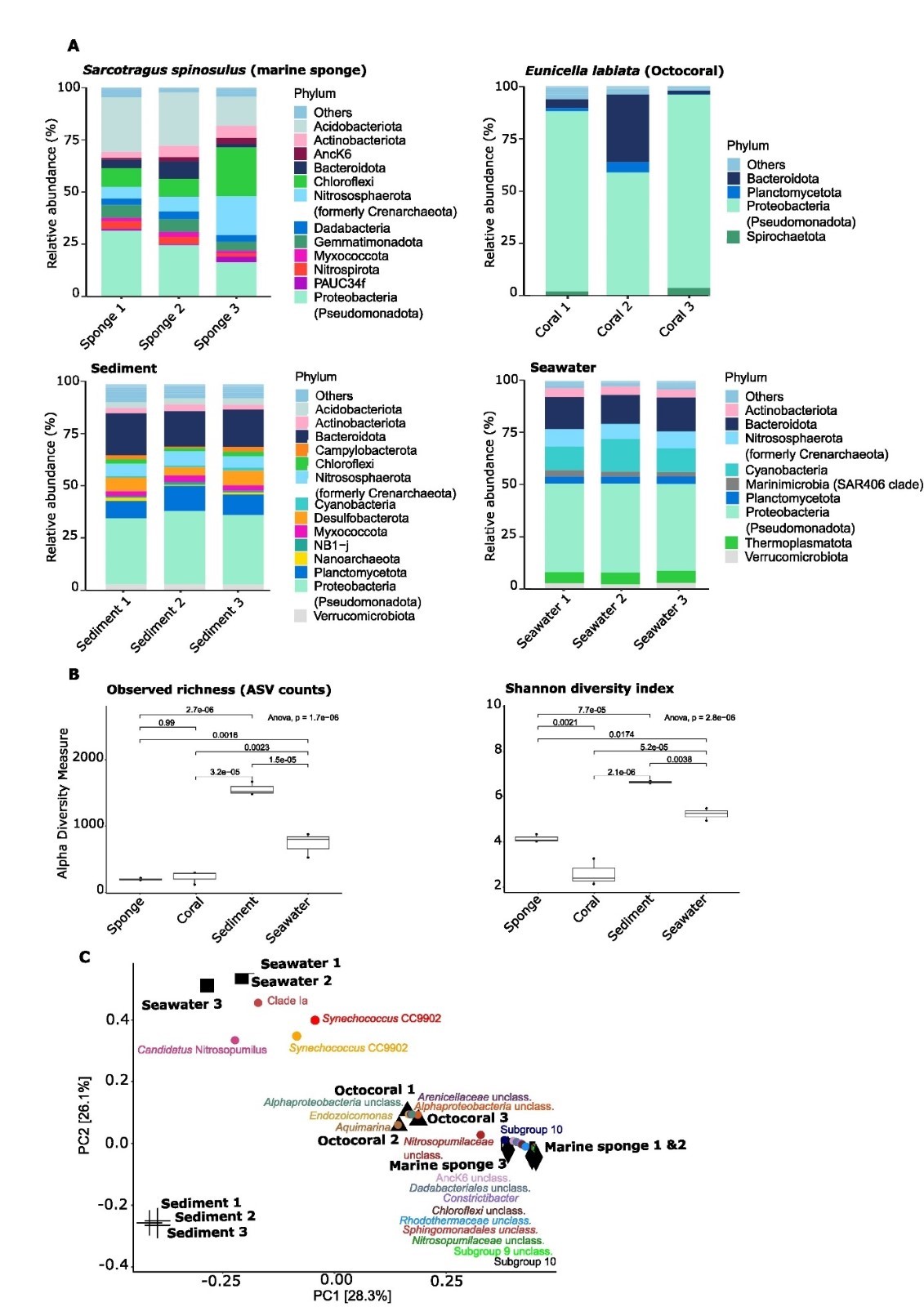


***Figure S2****: Prokaryotic communities (bacteria and archaea) in the four natural biotopes: the marine sponge Sarcotragus spinosulus, the octocoral Eunicella labiata and their surrounding seawater and sediment. A) Taxonomic composition at the phylum level. For each biotope, phyla whose relative abundances were lower than 1 % were merged into the category “Others”. B) Alpha diversity measures (Observed richness of ASVs and Shannon-Wiener diversity index). Boxes represent the interquartile range (IQR) between the first and third quartiles (25th and 75th percentiles, respectively), and the horizontal line inside the box defines the median. The p-values resulting from the ANOVA test and from the Tukey post-hoc tests are displayed on the graph. C) Principal coordinates analysis of prokaryotic communities from the different biotopes. Community ordination was performed on a Bray-Curtis similarity matrix calculated after Hellinger transformation of the ASV relative abundances. Samples are represented by black shapes (sediment – cross; seawater – square; marine sponge – diamond; octocoral - triangle). The 20 phylotypes (ASVs) that contribute the most to community dissimilarities among sample groups as revealed by SIMPER analyses were plotted in colored dots. Their position in the ordination diagram reflects their relative abundance across all samples: the closer a phylotype to any given sample, the higher its relative abundance in that sample.*

**
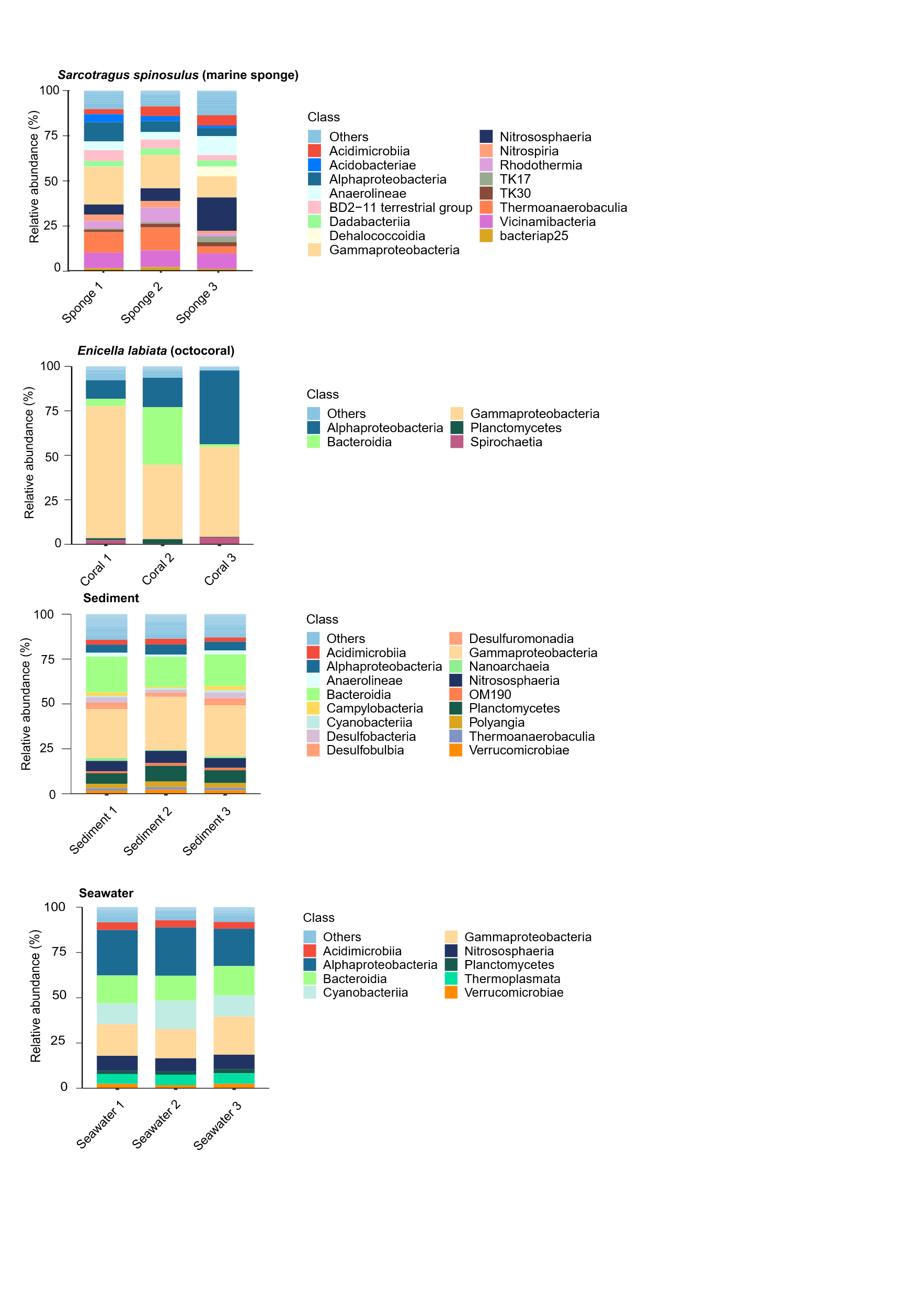
**

***Figure S3:*** *Class-level taxonomic composition of prokaryotic communities in environmental samples. In each biotope, the classes whose relative abundance was below 1 % were merged into the category “Others”.*


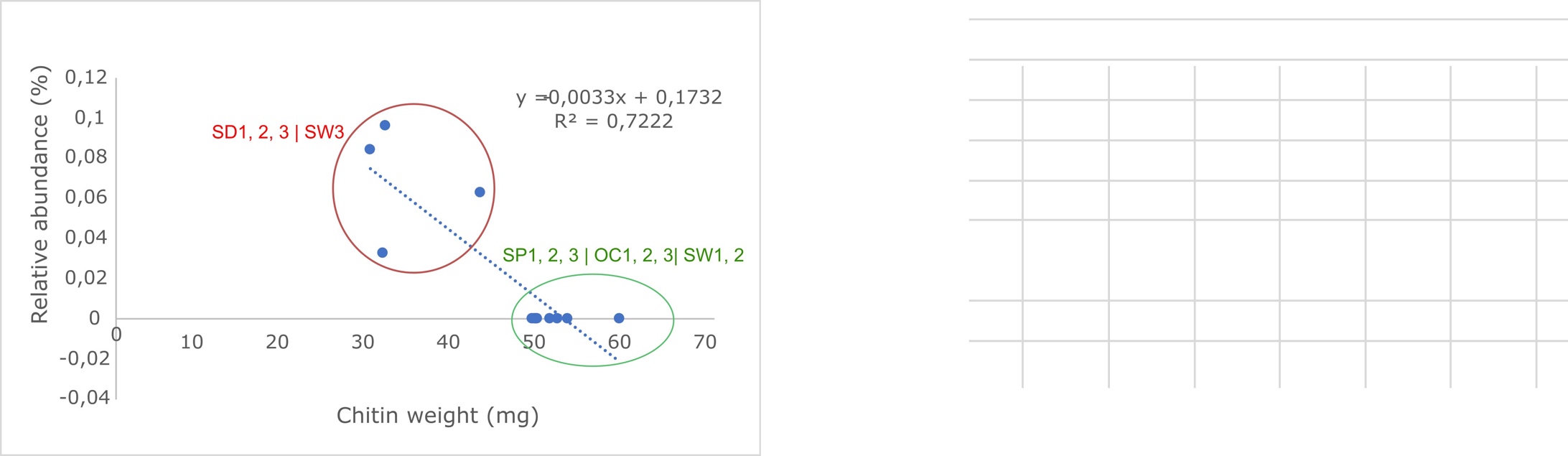


**Figure S4:** Relative abundance (%) of Psychromonas ASV26 in function of weight of remaining chitin (mg) in the culture 2 of each experiment. The samples for which chitin degradation was considered as good to high according to chitin weight and Mn1 measurements were surrounded by a red circle and the other ones (low to moderate chitin degradation) were surrounded by a green circle.

**Bibliography**

1. Keller-Costa T, Eriksson D, Gonçalves JMS, Gomes NCM, Lago-Lestón A, Costa R. The gorgonian coral *Eunicella labiata* hosts a distinct prokaryotic consortium amenable to cultivation. FEMS Microbiol Ecol. 2017;93:fix143.

2. Chang C, Chen S, Zhang L. Novel hydrogels prepared via direct dissolution of chitin at low temperature: structure and biocompatibility. J Mater Chem. 2011;21:3865.

3. Min B-M, Lee SW, Lim JN, You Y, Lee TS, Kang PH, et al. Chitin and chitosan nanofibers: electrospinning of chitin and deacetylation of chitin nanofibers. Polymer. 2004;45:7137–42.

4. Galed G, Diaz E, Goycoolea FM, Heras A. Influence of N-Deacetylation Conditions on Chitosan Production from α-Chitin. Nat Prod Commun. 2008;3:1934578X0800300414.

5. Yao K., Li J., Yao F., Yin Y. Chitosan-based hydrogels. CRC Press; 2012.

6. Kassambara A. ggpubr: ‘ggplot2’ Based Publication Ready Plots. GitHub. 2023. https://github.com/cran/ggpubr. Accessed 14 Apr 2023.

7. McMurdie PJ, Holmes S. phyloseq: An R Package for Reproducible Interactive Analysis and Graphics of Microbiome Census Data. PLoS ONE. 2013;8:1–11.

8. Wickham H, François R, Henry L, Müller K, Vaughan D, Software P, et al. dplyr: A Grammar of Data Manipulation. 2023. https://CRAN.R-project.org/package=dplyr. Accessed 15 Apr 2023.

9. Wickham H. ggplot2: Elegant Graphics for Data Analysis. Springer Int Publ. 2016.

10. Fox J, Weisberg S. cran/car. GitHub. 2023. https://github.com/cran/car. Accessed 14 Apr 2023.

11. Kassambara A. rstatix: Pipe-Friendly Framework for Basic Statistical Tests. GitHub. 2023. https://cran.r-project.org/web/packages/rstatix/index.html. Accessed 4 May 2023.

12. Oksanen J, Blanchet F, Kindt R, Legendre P, Minchin P. vegan: R package for community ecologists: popular ordination methods, ecological null models & diversity analysis. GitHub. 2013. https://github.com/vegandevs/vegan. Accessed 14 Apr 2023.

13. Hamidi B, Wallace K, Vasu C, Alekseyenko AV. Wd*-test: robust distance-based multivariate analysis of variance. Microbiome. 2019;7:51.

14. Russel J. GitHub - Russel88/MicEco: Various functions for analysis of microbial community data. GitHub. 2020. https://github.com/Russel88/MicEco. Accessed 13 Jul 2023.

15. Martinez Arbizu P. pairwiseAdonis. GitHub. 2020. https://github.com/pmartinezarbizu/pairwiseAdonis. Accessed 14 Apr 2023.

16. Hammer O, Harper DAT, Ryan PD. PAST: Paleontological Statistics Software Package for Education and Data Analysis. Palaeontol Electron. 2001;4:1.

17. Hardoim CCP, Costa R. Temporal dynamics of prokaryotic communities in the marine sponge *Sarcotragus spinosulus*. Mol Ecol. 2014;23:3097–112.

18. Boulotte NM, Dalton SJ, Carroll AG, Harrison PL, Putnam HM, Peplow LM, et al. Exploring the Symbiodinium rare biosphere provides evidence for symbiont switching in reef-building corals. ISME J. 2016;10:2693–701.

19. Ziegler M, Eguíluz VM, Duarte CM, Voolstra CR. Rare symbionts may contribute to the resilience of coral–algal assemblages. ISME J. 2018;12:161–72.

20. Podell S, Blanton JM, Neu A, Agarwal V, Biggs JS, Moore BS, et al. Pangenomic comparison of globally distributed *Poribacteria* associated with sponge hosts and marine particles. ISME J. 2019;13:468–81.

21. Oren A, Garrity GM. Valid publication of the names of forty-two phyla of prokaryotes. Int J Syst Evol Microbiol. 2021;71:005056.

22. Könneke M, Bernhard AE, de la Torre JR, Walker CB, Waterbury JB, Stahl DA. Isolation of an autotrophic ammonia-oxidizing marine archaeon. Nature. 2005;437:543–6.

23. Bayer K, Schmitt S, Hentschel U. Physiology, phylogeny and in situ evidence for bacterial and archaeal nitrifiers in the marine sponge *Aplysina aerophoba*. Environ Microbiol. 2008;10:2942–55.
